# Two receptor-targeting mechanisms of lambda-like siphophage Gifsy-1 of *Salmonella* Typhimurium

**DOI:** 10.1371/journal.ppat.1013352

**Published:** 2025-07-31

**Authors:** Xiaoli Zeng, Wenyu Wang, Dekang Zhu, Mafeng Liu, Mingshu Wang, Renyong Jia, Shun Chen, Qiao Yang, Ying Wu, Shaqiu Zhang, Juan Huang, Bin Tian, Xumin Ou, Di Sun, Yu He, Zhen Wu, Anchun Cheng, Xinxin Zhao

**Affiliations:** 1 Institute of Veterinary Medicine and Immunology, College of Veterinary Medicine, Sichuan Agricultural University, Chengdu, Sichuan, China; 2 Research Center of Avian Diseases, College of Veterinary Medicine, Sichuan Agricultural University, Chengdu, Sichuan, China; 3 Agricultural Animal Diseases and Veterinary Public Health Key Laboratory of Sichuan Province, Chengdu, Sichuan, China; 4 Engineering Research Center of Southwest Animal Disease Prevention and Control Technology, Ministry of Education of the People’s Republic of China, Chengdu, Sichuan, China; University of California Davis School of Medicine, UNITED STATES OF AMERICA

## Abstract

The receptor-targeting mechanisms by which λ-like siphophages establish infection in gram-negative bacteria remain poorly characterized. This study demonstrated that the λ-like phage Gifsy-1, which exhibits broad lytic activity in *Salmonella enterica*, employs two receptor-targeting mechanisms mediated by the side tail fiber Stf and central tail tip J dependent on O-polysaccharide (OPS) production. In rough (OPS-deficient LPS) *Salmonella* Typhimurium strains, Gifsy-1 employs multiple receptor-targeting: the J protein binds OmpC, OmpX, and BtuB, while the Stf protein targets galactose II (Gal II) of the lipopolysaccharide (LPS) core oligosaccharide. OmpC uniquely serves dual roles as the primary receptor (mediating initial adsorption) and secondary receptor (facilitating DNA ejection), whereas the other three receptors function exclusively as primary receptors to prompt high-efficiency phage adsorption. In contrast, the surface OPS in smooth *Salmonella* Typhimurium blocks J protein interactions with membrane proteins. Instead, Gifsy-1 utilizes core oligosaccharide Gal II, located between the OPS layer and outer membrane, as its necessary receptor for both adsorption and DNA ejection. This study intriguingly identified a shift in the receptor role of the core oligosaccharide in Gifsy-1 infection, which confers Gifsy-1 adaptation to OPS switching. The adaptability of the two targeting-mechanisms contributes to the understanding of the biological functions of Gifsy-1 and provides a blueprint for engineering phage therapy against multidrug-resistant *Salmonella enterica*.

## Author summary

The O-polysaccharide (OPS) of lipopolysaccharide (LPS) has a dual effect on bacterial susceptibility to phages, either preventing or promoting phage infections. *Salmonella enterica* strains switch OPS phases between smooth LPS and rough LPS for adaptation in natural environments. However, how phages adapt to bacterial OPS variations is elusive. This study demonstrated that phage Gifsy-1 of *Salmonella* Typhimurium deploys distinct receptor-targeting mechanisms to infect smooth and rough bacteria. OmpC is exploited by the Gifsy-1 central tail tip J when facing rough bacteria, while the OPS of smooth bacteria prevents phage recognition of OmpC. In this case, Gifsy-1 rather targets the conserved Gal Ⅱ moiety of the LPS core via the side tail fiber Stf. This feature grants Gifsy-1 the flexibility to infect both smooth and rough *Salmonella* Typhimurium.

## Introduction

Temperate phages are highly prevalent, with approximately 80% of bacteria in the murine gut microbiota being lysogens carrying integrated prophages [1,2]. These phages exert profound ecological and evolutionary influences on microbial ecosystems through horizontal gene transfer to disseminate virulence factors and metabolic genes that enhance host adaptation in animal niches [3–6].

Bacteriophage therapy is re-emerging as a critical solution for combating antibiotic-resistant bacterial infections, capitalizing on the innate capacity of phages for host-specific recognition and lysis [7,8]. Through synthetic biology modifications, engineered temperate phages are being employed as targeted therapeutic platforms, either by delivering antibacterial modules [9,10] or converting them to obligate lytic phages [11,12]. Optimizing phage cocktail therapy requires strategic formulations of phages with complementary receptor-targeting specificities [13]. Elucidating the molecular basis of the host-targeting mechanism of phages, which defines host specificity, is essential for understanding the roles played by phages in microbial evolution, ecology, and therapeutic applications [14].

The most well-studied phage types are the *Caudovirales* phages, which are classified into three families: *Podoviridae*, *Myoviridae*, and *Siphoviridae*. The infection of tailed phages is generally initiated with reversible adsorption to a first “primary” receptor on host cells, which is optional or essential for the subsequent step of irreversible binding to a “secondary” receptor to eject phage DNA [14]. They typically use tail proteins (tail fibers, tail spikes, and tail tips) as receptor-binding proteins (RBPs) to interact with bacterial receptors. Nearly all bacterial surface components can be exploited by phages as receptors, including surface polysaccharides (e.g., lipopolysaccharide (LPS), capsules, and enterobacterial common antigen (ECA)), flagella, teichoic acids, and a variety of outer membrane proteins (OMPs) [15], with LPS being the main receptor in gram-negative bacteria [14].

Intact (smooth) LPS, a major component of the outer membrane (OM), is composed of O-polysaccharide (OPS), core oligosaccharide (COS), and lipid A, whereas rough LPS lacks OPS [16]. OPS, the outermost component of LPS, acts as a mechanical barrier that shields the OM from various molecules, such as bacteriocins, antibodies, and phages [17]. In a BASEL coliphage collection study, 70% of the coliphages were either completely or significantly inhibited by O16-type OPS in *E. coli*. However, all of these coliphages can lyse the rough *E. coli* strain by targeting its COS, OMPs, residual OPS glycan, or ECA [18]. The lytic siphophage SSU5 can infect only rough *Salmonella enterica* (*S. enterica*) strains by binding to the COS region with the tail fiber gp22 [19]. Phages have evolved mechanisms to overcome the OPS barrier. Podophages, such as *Salmonella* phage P22 and coliphage G7C, penetrate the OPS layer through tail spikes with enzymatic activities (depolymerization or deacetylation), which are essential for accessing secondary OM receptors [20,21]. Myophages equipped with contractile tails, such as coliphage T4, trigger baseplate conformational changes upon receptor recognition of LPS or OMPs by long tail fibers [22]. Baseplate rearrangement and expansion facilitate the mechanical engagement of short tail fibers, enabling them to penetrate the outer OPS layer and irreversibly bind secondary receptors [23].

Host recognition mechanisms of siphophages with long and noncontractile tails have been insufficiently characterized. The siphophage for which the early infection mechanism has been well described is the coliphage T5. T5 initiates infection by adsorption on the OPS of smooth LPS via lateral tail fibers, which facilitates the recognition of the outer membrane receptor BtuB by central tail fibers [24]. Siphophage λ infection is blocked by the OPS of *E. coli* [25], which lyses the rough strain by targeting the receptor LamB via the central tail tip J [26]. The temperate λ-like siphophage Gifsy-1 exhibits greater plating efﬁciency and larger plaques in the *galE* mutant strain (lacking the outer core and OPS) than in wild-type (WT) *S. enterica* serovar Typhimurium (*S.* Typhimurium) [27]. The OmpC of the rough strain is exploited by Gifsy-1 for adsorption and plaque formation [27]; however, the receptor-targeting mechanisms of Gifsy-1 or other λ-like siphophages on smooth bacteria remain elusive [28].

Gifsy-1 prophage genes are crucial for the stress adaptation mechanisms of *S.* Typhimurium, maintaining bacterial genome integrity and ensuring the survival of *S. enterica* within animal hosts [29,30]. A recent epidemiological study revealed that Gifsy-1 is the most prevalent intact prophage in 303 *Salmonella* spp. genomes, encompassing 254 unique serovars [31]. This widespread distribution implies that Gifsy-1 has a broad host range and a specific strategy to overcome the OPS shield in smooth bacteria. In this study, we elucidated the receptor-targeting mechanisms of Gifsy-1 in smooth and rough *S.* Typhimurium strains. Notably, Gifsy-1 infection of the smooth strain was found to be independent of OmpC. Gifsy-1 possesses two distinct receptor-targeting mechanisms, and which strategy is employed depends on whether the smooth or rough LPS phenotype is displayed by *S*. Typhimurium.

## Results

### Determination of the host range of Gifsy-1

The lytic activities of the phage Gifsy-1 against *S. enterica* strains were first measured. Gifsy-1 was induced and isolated from the *S.* Typhimurium ATCC14028 derivative strain SLT40, whose prophage Gifsy-3 was cured. A phage solution from the control strain SLT41, which was cured with both Gifsy-1 and Gifsy-3, was used to determine whether bacterial lysis was specifically caused by Gifsy-1 rather than other phages in ATCC14028. Plaque formation by Gifsy-1 was tested on 171 WT *S. enterica* strains with 5 serogroups. A total of 44.44% of the smooth bacterial strains were susceptible to Gifsy-1, which significantly lysed four O serotypes of *S. enterica*, including serogroups B (43.40%), C2 (16.67%), D1 (84.48%), and K (14.29%), but not the C1 serotype (Tables 1 and S1). Therefore, Gifsy-1 exhibited a broad host range for *S. enterica* and the ability to infect smooth *S. enterica* strains with at least four serotypes of OPS. To reveal the host recognition mechanism of Gifsy-1, the WT *S.* Typhimurium strain S-13 was used as an indicator of susceptibility in subsequent experiments.

**Table 1:** Host range of Gifsy-1 in *S. enterica* strains.

Species	Serovars	Serogroups	Tested strains	Susceptible strains	Susceptible rate (%)
*S.* e*nterica*	Typhimurium	B	53	23	43.40
	Montevideo; Potsdam; Thompson; Choleraesuis	C1	45	0	0
	Kottbus; Newport	C2	6	1	16.67
	Enteritidis; Pullorum	D1	60	51	84.48
	Cerro	K	7	1	14.29

### Roles of OPS and OmpC of *S*. Typhimurium in Gifsy-1 infection

**Fig 1:**
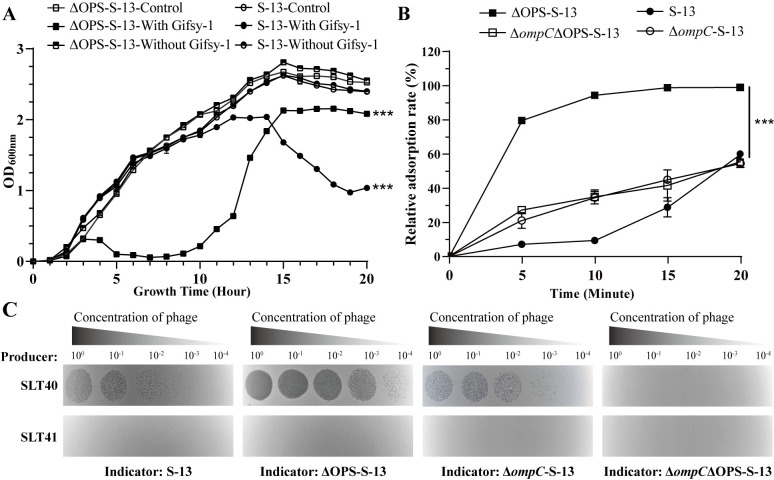
Determination of the effects of OPS and OmpC on phage Gifsy-1 infection. (A) Growth curves of the WT smooth strain S-13 and the rough strain ΔOPS-S-13 cultured alone or incubated with phages isolated from SLT40 (curing of Gifsy-3) or SLT41 (curing of Gifsy-1 and Gifsy-3). **(B)** Adsorption curves of Gifsy-1 on S-13, ΔOPS-S-13, Δ*ompC*ΔOPS-S-13, and Δ*ompC*-S-13. **(C)** EOP of phage isolates from SLT40 or SLT41 on S-13, ΔOPS-S-13, Δ*ompC*ΔOPS-S-13, and Δ*ompC*-S-13. A paired *t* test followed by two-tailed calculation was used to determine differences between groups. The asterisk next to the error bar indicates a significant difference compared with the control group. The asterisk next to the line indicates a signiﬁcant difference between the two indicated groups. ***, **p* *< 0.001.

To identify the influence of OPS on Gifsy-1 infection, isolated Gifsy-1 phages were incubated with both the WT strain S-13 and its OPS-deficient mutant ΔOPS-S-13 (Δ*rmlB-rfbP*-S-13), followed by assessment of lysis curves, adsorption rates, and efficiency of plaque formation (EOP). S-13 and ΔOPS-S-13 showed similar growth curves when they were cultured alone or with the phage solution from SLT41 (ΔGifsy-1ΔGifsy-3) (Fig 1A). Both strains exhibited significant lysis after incubation with phage isolates from SLT40 (ΔGifsy-3) (Fig 1A), confirming that Gifsy-1 specifically killed S. Typhimurium. Compared with the smooth WT S-13, the rough variant ΔOPS-S-13 exhibited increased susceptibility to Gifsy-1, with accelerated and intensified lysis (Fig 1A), increased adsorption rates (Fig 1B), and elevated EOP (Fig 1C). This observation revealed that OPS impedes Gifsy-1 adsorption and DNA ejection, acting as a partial barrier to infection.

OmpC has been identified as a receptor that facilitates Gifsy-1 adsorption and DNA ejection into rough *S.* Typhimurium [27]. To further elucidate the role of OmpC in phage infection in the smooth and rough strains, we deleted the *ompC* gene from both the S-13 and ΔOPS-S-13 strains and measured the resulting phage infection efficiencies. Compared with that on the parental ΔOPS-S-13 strain, Gifsy-1 adsorption on the Δ*ompC*ΔOPS-S-13 strain was markedly reduced but not abolished (Fig 1B), indicating that OmpC is a primary receptor for Gifsy-1 adsorption on rough bacteria but not the sole receptor. In contrast, the complete absence of plaque formation on Δ*ompC*ΔOPS-S-13 confirmed its essential role as a secondary receptor in the context of rough LPS (Fig 1C). Furthermore, no significant differences in either phage adsorption at 20 min or EOP were observed between the S-13 and Δ*ompC*-S-13 strains (Fig 1B and 1C), indicating that OmpC was not involved in Gifsy-1 infection of smooth *S.* Typhimurium. These findings demonstrated that, in the smooth strain, the OPS of *S.* Typhimurium prevents Gifsy-1 from recognizing OmpC, implying that alternative membrane factors are exploited to promote phage infection.

### Role of COS in Gifsy-1 infection

**Fig 2:**
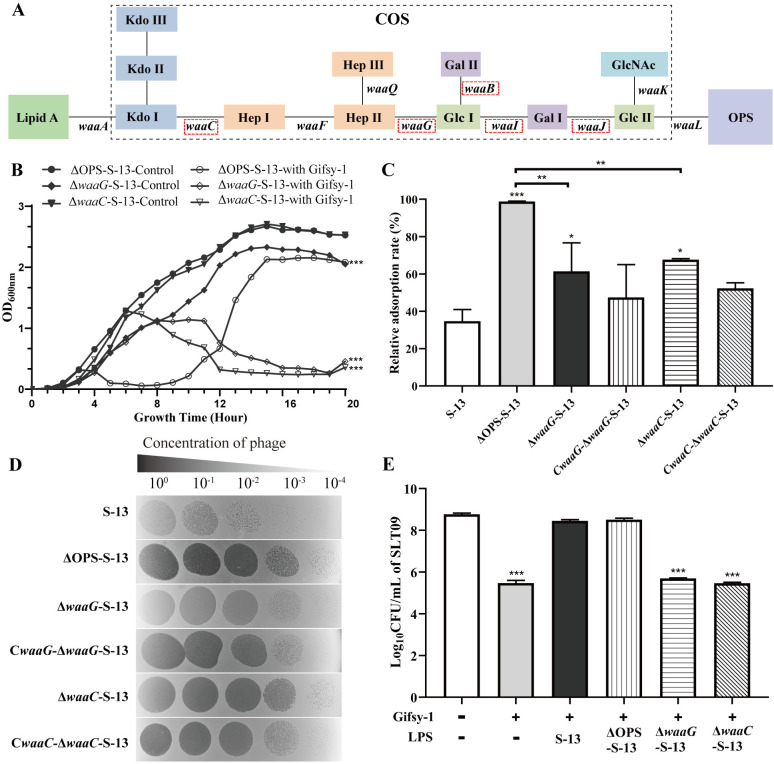
Identification of the role of COS in Gifsy-1 infection. (A) Schematic diagram of the structure and synthesis genes of the LPS COS of S. Typhimurium. Kdo, 3-deoxy-D-mannooctulo-sonic acid; Hep, Heptose; GlcNAc, N-acetylglucosamine; Glc, glucose; Gal, galactose; OPS, O-polysaccharide. The genes with red dashed lines were targeted for deletion. (B) Growth curves of ΔOPS-S-13, Δ*waaG*-S-13, and Δ*waaC*-S-13 cultured alone or incubated with the Gifsy-1. A paired *t* test followed by a two-tailed calculation was used to detect the difference of growth or adsorption curves of the groups. **(C-D)** Detection of the adsorption rates (C) and EOP (D) of Gifsy-1 on the S-13, ΔOPS-S-13, Δ*waaG*-S-13, Δ*waaC*-S-13, and complemented strains. **(E)** Lysis inhibition assays. After preincubation with LPS extracted from the WT S-13, ΔOPS-S-13, Δ*waaG*-S-13, and Δ*waaC*-S-13 strains, Gifsy-1 lysis on the ΔOPS-S-13 was measured. Differences between groups were analyzed by one-way ANOVA followed by Tukey’s multiple comparison test. The asterisk above the error bar indicates a significant difference compared with the leftmost group. The asterisk above the line indicates a signiﬁcant difference between the two indicated groups. *, *p* < 0.05; **, *p* < 0.01; ***, *p* < 0.001.

Because COS is located adjacent to the OPS and serves as a receptor for some phages, such as the siphophage SSU5 and myophage Bp7 [19,32], its role in Gifsy-1 infection was investigated. The *S*. Typhimurium COS consists of glycosyl groups formed by glycosyltransferases (Fig 2A) [33]. The OPS is structurally connected to the COS component. Deletion of the main-chain COS genes (e.g., *waaJ* and *waaI*) abolishes both COS and OPS, while side-chain COS deletions (e.g., *waaB* and *waaQ*) alter only COS without affecting OPS. We first deleted the *waaC* and *waaG* genes, which are responsible for heptose I (Hep I) and glucosyl I (Glc I) addition, respectively, from the S-13 strain. The Δ*waaG*-S-13 and Δ*waaC*-S-13 mutants presented rough LPS with truncated COS as shown by silver staining (S1 Fig). The two rough mutants presented delayed lysis (Fig 2B) and significantly lower adsorption rates upon Gifsy-1 infection than did ΔOPS-S-13 (Fig 2C). Complementation of *waaG* and *waaC in trans* partially restored the adsorption phenotypes (Fig 2C). Moreover, the deletion of *waaG* or *waaC* had a slight or negligible effect on plaque formation (Fig 2D). Therefore, in rough *S.* Typhimurium strains, COS functions as a primary receptor for Gifsy-1 adsorption but is irrelevant to Gifsy-1 DNA ejection.

Phage lysis inhibition assays with LPS from S-13 and its derivative mutants were further conducted to assess the COS-Gifsy-1 interaction. Gifsy-1 exhibited strong lytic activity against ΔOPS-S-13, which was abrogated by the addition of LPS from the WT (intact LPS) or ΔOPS-S-13 (COS + lipid A) strain (Fig 2E). In contrast, LPS from the Δ**waa*G*-S-13 (Hep × 3 + Kdo × 2 + lipid A) or Δ*waaC*-S-13 (Kdo × 2 + lipid A) mutant failed to inhibit Gifsy-1 lysis, indicating that Gifsy-1 bound the COS of *S*. Typhimurium at a site between Hep II and OPS.

### Determination of the COS moiety recognized by Gifsy-1

**Fig 3:**
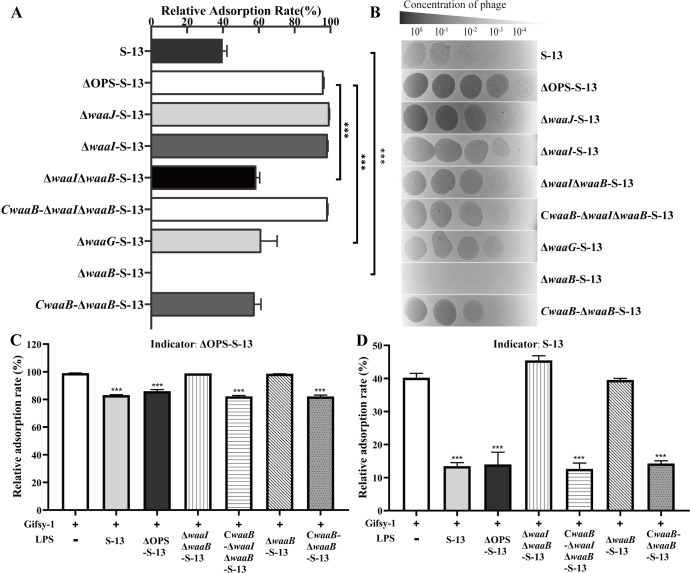
Identification of the COS moiety exploited by Gifsy-1 via the detection of phage infections. (A-B) Detection of the adsorption rates and EOP of Gifsy-1 on S. Typhimurium strains, including the WT S-13, ΔOPS-S-13 and various COS mutant strains and complemented strains. **(C-D)** Adsorption inhibition assays by LPS. Gifsy-1 was incubated with LPS extracted from the WT S-13, ΔOPS-S-13, Δ*waaI*Δ*waaB*-S-13, Δ*waaB*-S-13 or the related complemented strains. Then, phage adsorption on ΔOPS-S-13 (C) or S-13 (D) was measured. Differences between groups were analyzed by one-way ANOVA followed by Tukey’s multiple comparison test. The asterisk above the error bar indicates a difference compared with the leftmost group. The asterisk above the line indicates a signiﬁcant difference between the two indicated groups. ***, *p* < 0.001.

To identify the Gifsy-1 recognition site on COS, we generated three rough mutants: Δ*waaJ*-S-13, Δ*waaI*-S-13, and Δ*waaI*Δ*waaB*-S-13. These mutants were defective in OPS production and the transfer of Glc Ⅱ, galactose Ⅰ (Gal Ⅰ), and both Gal Ⅰ and Gal Ⅱ, respectively (Fig 2A). A smooth mutant Δ**wa*aB*-S-13 was also constructed, which retained smooth LPS synthesis but lacked only a single Gal Ⅱ residue in the COS (Fig 2A), as confirmed by silver-staining analysis (S1 Fig). Adsorption assays revealed that the rough mutant Δ**wa*aJ*-S-13 or Δ*waaI*-S-13 had no difference on the adsorption of Gifsy-1 compared with ΔOPS-S-13 (Fig 3A). In contrast, the Δ*waaI*Δ*waaB*-S-13 rough mutant presented a significantly reduced adsorption rate, similar to that of Δ*waaG*-S-13. Complementation of *waaB in trans* in Δ*waaI*Δ*waaB*-S-13 restored the adsorption phenotype (Fig 3A). The EOP of Δ**waa*J*-S-13, Δ*waaI*-S-13, and Δ*waaI*Δ*waaB*-S-13 were only slightly lower than those of ΔOPS-S-13 (Fig 3B). Surprisingly, Gifsy-1 thoroughly failed to adsorb or form plaques on the smooth Δ**waa*B*-S-13 strain, whereas it efficiently lysed the WT strain. Complementation of *waaB in trans* in the Δ*waaB*-S-13 strain restored both the adsorption and plaque formation phenotypes (Fig 3A and 3B). Interestingly, these findings demonstrated that Gifsy-1 recognizes the branched Gal Ⅱ moiety of COS and that Gal Ⅱ has a dual role in Gifsy-1 infection. Gal Ⅱ acts as one of the primary receptors for phage adsorption on the rough *S.* Typhimurium strain while serves as both primary and secondary receptor exploited by the phage to complete infection of the smooth *S.* Typhimurium strain.

Phage lysis and adsorption inhibition experiments were performed to determine the interaction between the COS Gal Ⅱ and Gifsy-1. LPS from the S-13 and ΔOPS-S-13 strains strongly inhibited Gifsy-1 lysis (S2 Fig) and adsorption (Fig 3C and 3D) on both ΔOPS-S-13 and S-13, with a notably greater reduction in adsorption on S-13 relative to ΔOPS-S-13. Nevertheless, LPS lacking Gal II, from either Δ*waaI*Δ*waaB*-S-13 or Δ*waaB*-S-13, inhibited neither phage lysis (S2 Fig) nor adsorption (Fig 3C and 3D) on both ΔOPS-S-13 and S-13; complementation of *waaB* in the two mutants restored the inhibitory effects of LPS.

### Identification of phage RBPs for recognizing smooth or rough *S*. Typhimurium

**Fig 4:**
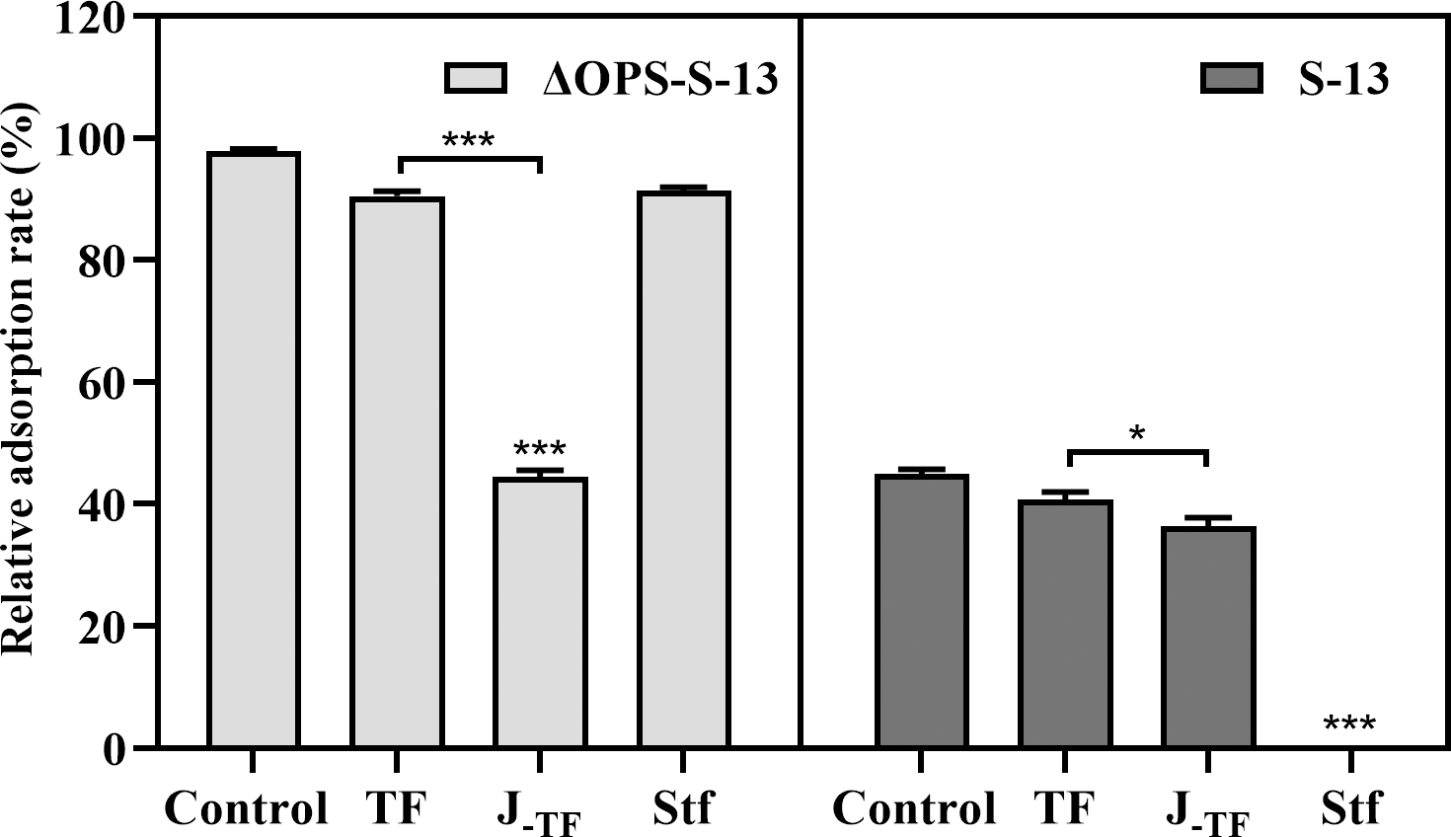
Characterization of RBPs of Gifsy-1 via an adsorption inhibition assay. The S. Typhimurium strains ΔOPS-S-13 and S-13 were preincubated with purified TF, J_-TF_, Stf, or the protein solution control, respectively. Then, the mixture was infected with the Gifsy-1 at an MOI of 0.01, followed by measurement of the relative adsorption rates. Differences between groups were determined by two-way ANOVA followed by Tukey’s multiple comparison test. The asterisk above the error bar indicates a significant difference compared with the control. The asterisk above the line indicates a signiﬁcant difference between the two indicated groups. *, *p* < 0.05; ***, *p* < 0.001.

In siphophages, RBPs typically manifest as tail fibers. Gifsy-1 is equipped with the central tail tip J (GenBank: WP_138930548.1) and the side tail fiber Stf (WP_001144691.1). Protein J shared 70.61% identity with its λ phage orthologue, with 78.88% sequence identity in the N-terminal region but only 18% in the C-terminal region, a domain essential for receptor binding in λ phage [34,35] (S3A Fig). Stf shared 50.65% identity with its Ur-λ phage orthologue (S3B Fig), which is functional and targets OmpC [36]. To confirm the roles of the J and Stf fibers, purified proteins were preincubated with smooth or rough *S*. Typhimurium strains, and the adsorption of Gifsy-1 was subsequently measured. Interestingly, compared with the solubility-enhancing TF tag control, the purified protein J_-TF_ significantly reduced phage adsorption on the rough ΔOPS-S-13 strain, whereas Stf had no adverse effects (Fig 4). Conversely, for the smooth S-13 strain, Stf completely inhibited phage adsorption, whereas J caused only a slight decrease (Fig 4). These findings indicated that Gifsy-1 primarily utilizes Stf for recognizing the smooth strain and J for recognizing the rough strain of *S*. Typhimurium.

### Identification of all primary receptors for Gifsy-1 that target rough *S*. Typhimurium

**Fig 5:**
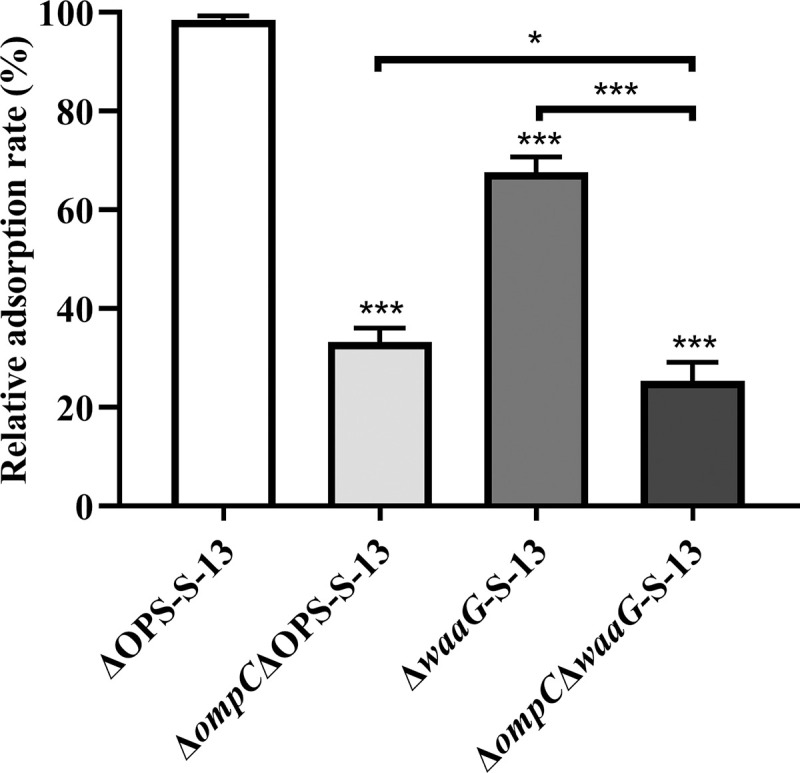
Detection of phage adsorption on rough S. Typhimurium strains. Bacterial strains including the ΔOPS-S-13, Δ*ompC*ΔOPS-S-13, Δ*waaG*-S-13 and Δ*waaG*Δ*ompC*-S-13 were incubated with Gifsy-1 at an MOI of 0.01, respectively. Then, the relative adsorption rates were calculated in each group. Differences between groups were determined by one-way ANOVA followed by Tukey’s multiple comparison test. The asterisk above the error bar indicates a difference compared with the ΔOPS-S-13 group. The asterisk above the line indicates a signiﬁcant difference between the two indicated groups. *, *p* < 0.05; ***, *p* < 0.001.

Our study confirmed that Gifsy-1 relies on OmpC and Gal Ⅱ of COS for adsorption on rough *S*. Typhimurium strains. Further investigation into potential additional primary receptors was conducted by examining phage adsorption on the Δ*ompC*Δ**wa*aG*-S-13 strain. Compared with ΔOPS-S-13, strains lacking either *ompC* or *waaG* exhibited notable decreases in adsorption; consequently, the simultaneous loss of both genes resulted in an even greater reduction (Fig 5). Nonetheless, Gifsy-1 still maintained 25% of its ability to adsorb on the Δ*ompC*Δ*waaG*-S-13 strain (Fig 5), suggesting the presence of other alternative primary receptors that promote Gifsy-1 adsorption on rough strains.

**Fig 6:**
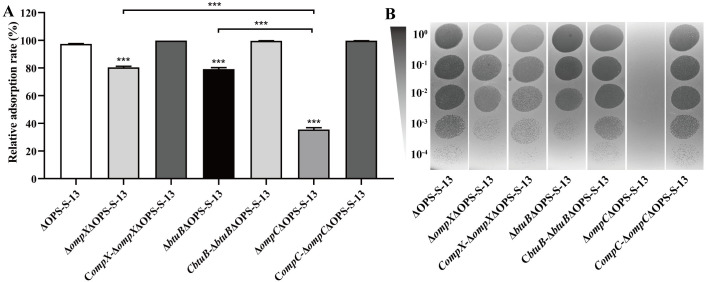
Identification of protein receptors involved in Gifsy-1 infection of rough S. Typhimurium strains. The membrane proteins estimated to be targeted by Gifsy-1 were deleted from the ΔOPS-S-13. The phage adsorption **(A)**, and EOP (B) of Gifsy-1 were assayed on the ΔOPS-S-13 and its derived mutants, and some complemented strains. Differences between groups were analyzed by one-way ANOVA followed by Tukey’s multiple comparison test. The asterisk above the error bar indicates a difference compared with the ΔOPS-S-13 group. The asterisk above the line indicates a signiﬁcant difference between the two indicated groups. ***, *p* < 0.001.

To identify other primary receptors, a pull-down assay with the J_-His_ protein against membrane proteins of *S*. Typhimurium was conducted, followed by protein A/G affinity chromatography, SDS‒PAGE, and peptide mass spectrometry. This method identified seven OM proteins: OmpX, OmpD, OmpV, FepA, Tsx, CirA, and BtuB. The effects of these proteins and another three phage OM receptors (FhuA, TolB, and FlgH) on phage activity were subsequently examined. Gifsy-1 lysis assays indicated that the deletion of *ompX*, *btuB*, or *ompC* restored rough strain ΔOPS-S-13 growth from inhibition (S4A Fig). In addition to the Δ*ompC*ΔOPS-S-13 mutant, the Δ*ompX*ΔOPS-S-13 and Δ*btuB*ΔOPS-S-13 mutants also exhibited decreased susceptibility to Gifsy-1 adsorption relative to the parent strain (Fig 6A), whereas the deletion of other genes had negligible effects (S4B Fig). Trans-complementation of *ompX*, *btuB*, or *ompC* restored the phage susceptibility of the bacteria (Fig 6A). Moreover, deletion of *ompC* abolished plaque formation, whereas *ompX* or *btuB* deletion induced only a minor reduction in plaque formation (Fig 6B). Loss of *tolB* or *tsx* led to an approximately 10- or 100-fold reduction in the EOP (S4C Fig), suggesting that the two OMPs participate in the process of DNA ejection of Gifsy-1 in a cooperative manner with OmpC.

**Fig 7:**
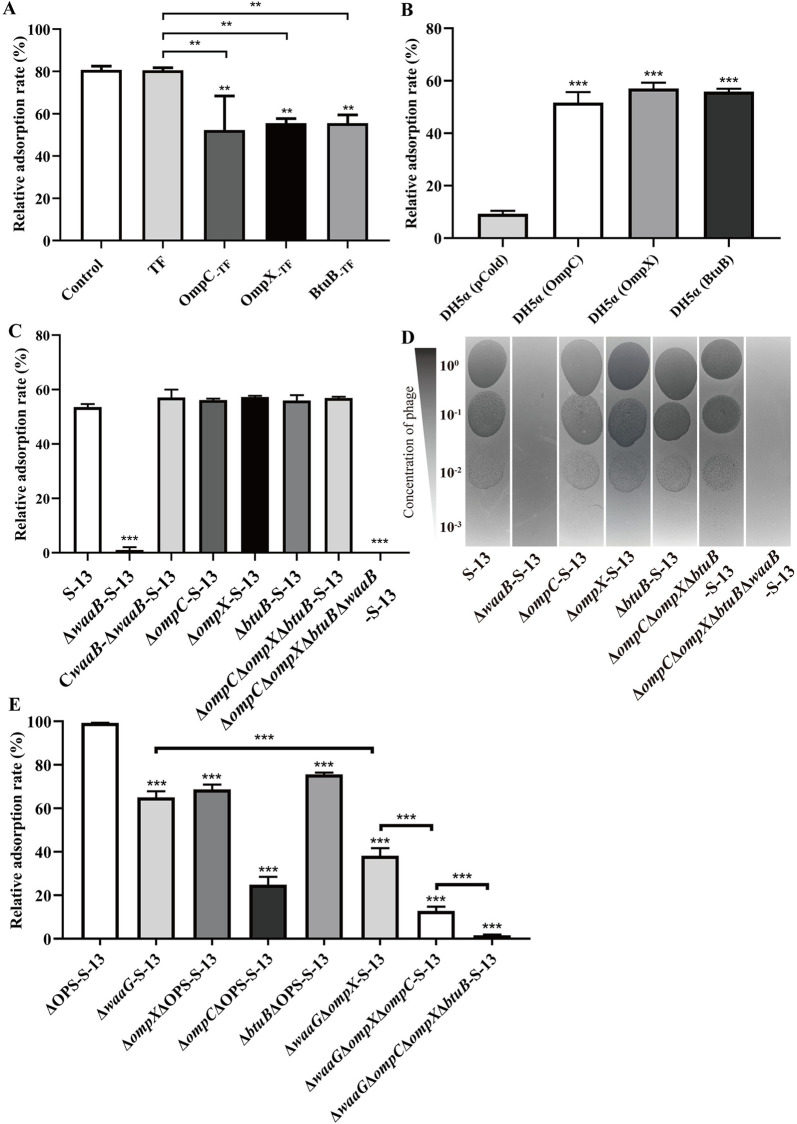
Determination of the roles of Gifsy-1 membrane receptors in phage adsorption and plaque formation. (A) Phage adsorption inhibition assays. Gifsy-1 was preincubated with purified TF, OmpC_-TF_, OmpX_-TF_, BtuB_-TF_, or the protein solution control. The mixture was then subjected to phage adsorption assay on the ΔOPS-S-13 strain. (B) Detection of phage adsorption on the *E. coli* DH5α and *E. coli* strains with recombinant expression of OmpC, OmpX, or BtuB from S. Typhimurium. (C-D) Detection of the adsorption rates (C) and EOP (D) of Gifsy-1 on smooth S. Typhimurium strains, including the WT S-13 strain and related mutants. (E) Detection of phage adsorption on various S. Typhimurium mutant strains with deletion of four primary receptors of Gifsy-1. Differences between groups were analyzed by one-way ANOVA followed by Tukey’s multiple comparison test. The asterisk above the error bar indicates a difference compared with the leftmost group. **, *p* < 0.01; ***, *p* < 0.001.

Compared with the TF tag control, preincubation with OmpX_-TF_, BtuB_-TF_ or OmpC_-TF_ significantly reduced the adsorption rate of Gifsy-1 on the ΔOPS-S-13 strain (Fig 7A). The *E. coli* strain DH5α was refractory to Gifsy-1 adsorption; however, heterologous expression of OmpC, OmpX, or BtuB from *S.* Typhimurium in DH5α rendered the bacterium highly vulnerable (Fig 7B). Thus, OmpX and BtuB, in addition to OmpC and COS Gal Ⅱ, serve as primary receptors for Gifsy-1 during infection of rough *S*. Typhimurium. Next, to observe whether these protein receptors are involved in the recognition of smooth *S.* Typhimurium, the *ompX* or *btuB* gene was deleted from the S-13 strain. As described above, Gifsy-1 lost the ability to adsorb onto or form plaques in Δ*waaB-*S-13; however, neither individual nor combined deletions of *ompX, btuB,* and *ompC* affected adsorption (Fig 7C) and plaque formation (Fig 7D) in the smooth strain S-13. In contrast, the deletion of *waaB* in Δ*ompX*Δ*btuB*Δ**om*pC*-S-13 background abolished phage adsorption (Fig 7C) and plaque formation (Fig 7D), confirming the role of COS Gal Ⅱ as the sole receptor for Gifsy-1 infection of the smooth strain.

Additionally, we evaluated the contributions and relationships of four primary receptors (OmpC, OmpX, BtuB, and COS) involved in Gifsy-1 adsorption onto rough *S*. Typhimurium. Individual deletions of these receptors impaired phage adsorption, with *ompC* deletion causing the most significant reduction (Fig 7E). Progressive deletion of *ompX*, *ompC*, and *btuB* in the Δ*waaG*-S-13 strain led to a sustained decline in adsorption, which ceased when all four receptors were absent (Fig 7E). Therefore, these four receptors act as coreceptors of Gifsy-1, facilitating phage adsorption on rough *S*. Typhimurium, with OmpC emerging as the most pivotal.

### Determination of interactions between RBPs and multiple receptors

**Fig 8:**
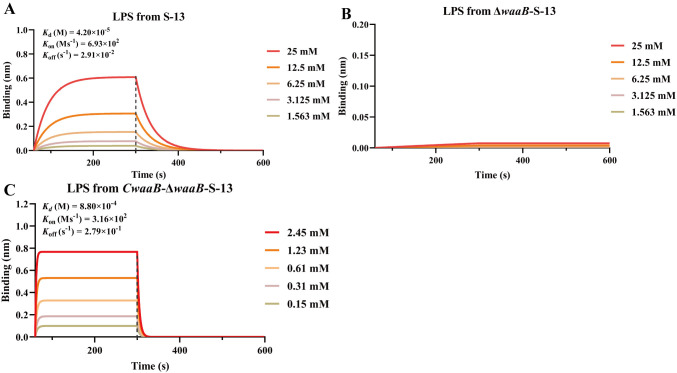
Determination of interactions between the Stf protein and S. Typhimurium LPS via the BLI assay. The protein Stf was tethered to the ProA sensor, and the kinetics of Stf binding different concentrations of LPS extracted from the S-13 strain **(A)**, the Δ*waaB*-S-13 strain **(B)**, or the complemented strain *CwaaB*-Δ*waaB*-S-13 (C) were measured and were shown as binding profiles. The left and right sides of the dotted line correspond to the association and dissociation curves, respectively. *K*_d_: equilibrium dissociation constant; *K*_on_: association rate constant; *K*_off_: dissociation rate constant.

Our findings revealed two RBPs of Gifsy-1 (J and Stf) and multiple receptors exploited by Gifsy-1 to recognize *S*. Typhimurium strains. A biolayer interferometry (BLI) assay was utilized to elucidate the interaction between Stf and the exclusive receptor of smooth bacteria, the COS Gal Ⅱ. A binding profile revealed that S-13 LPS bound Stf directly, with an equilibrium dissociation constant (*K*_d_) of 4.2 × 10^-5^ M (Fig 8A). In contrast, the LPS from the Δ**waa*B*-S-13 strain did not bind Stf (Fig 8B), indicating that the binding site of Stf on COS was Gal Ⅱ. Trans-complementation of *waaB* in Δ**wa*aB*-S-13 restored the ability of LPS to bind Stf, with an equilibrium dissociation constant of 8.8 × 10^-4^ M (Fig 8C). The association and dissociation of LPS from the complementary strain to Stf were faster than those of the LPS from WT S-13 (Fig 8A and 8C).

The other RBP of Gifsy-1, tail tip J, was fused with GFP to assess its interactions with bacterial receptors, and the bacteria that were fluorescently labelled with GFP-J were quantified via spectrofluorometry. High fluorescence intensity was observed following incubation of GFP-J with the ΔOPS-S-13 and Δ*waaG*-S-13 strains, but significant decreases were noted with S-13 and the mutants Δ*ompC*ΔOPS-S-13, Δ*ompX*ΔOPS-S-13, and Δ*btuB*ΔOPS-S-13 (S5 Fig). Deletion of *ompC* caused the most significant decrease in fluorescence intensity, followed by *btuB* and *ompX* deletions, with the latter resulting in the weakest effect. Complementation of *ompC*, *btuB*, or *ompX* in the mutant strain restored fluorescence labelling (S5 Fig). Extremely low fluorescence was detected after the incubation of GFP with any of these strains (S5 Fig). These findings indicated that the J protein specifically binds the OMP receptors OmpC, BtuB, and OmpX rather than COS.

**Fig 9:**
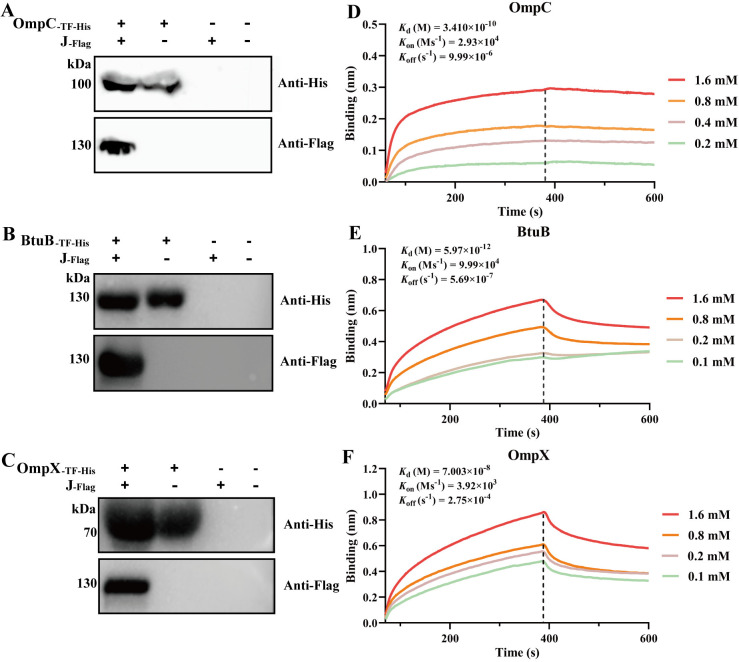
Determination of interactions between the J protein and three OMPs. (A-C) Detection of the protein interactions of J_-Flag_ with OmpC_-TF-His_ (A), BtuB_-TF-His_ (B), or OmpX_-TF-His_ (C) via pull-down assays and western blotting with a His_6_ mAb and a Flag mAb. (D-F) Detection of BLI response profiles of the different concentrations of J protein bound to the OmpC (D), BtuB (E), or OmpX (F).

To further examine the direct interactions of the tail tip J and protein receptors, pull-down and BLI assays were performed. Pull-down assays demonstrated the strong and specific binding of J_-Flag_ to OmpC_-TF-His_, BtuB_-TF-His_, and OmpX-_TF-His_, respectively (Fig 9A-9C). The tag TF did not interact with J_-Flag_ (S6 Fig). The BLI response profile revealed distinct affinities of the three OMPs for the J protein, with BtuB exhibiting the strongest interaction (*K*_d_ values: OmpC, 3.41 × 10^-10^ M; OmpX, 7.003 × 10^-8^ M; BtuB, 5.97 × 10^-12^ M) (Fig 9D-9F). Moreover, the J protein exhibited highly stable and irreversible binding with OmpC, as evidenced by a minimal decrease in the slope of the dissociation curve (Fig 9D).

### Two receptor-targeting mechanisms of the phage Gifsy-1 in *S*. Typhimurium

**Fig 10:**
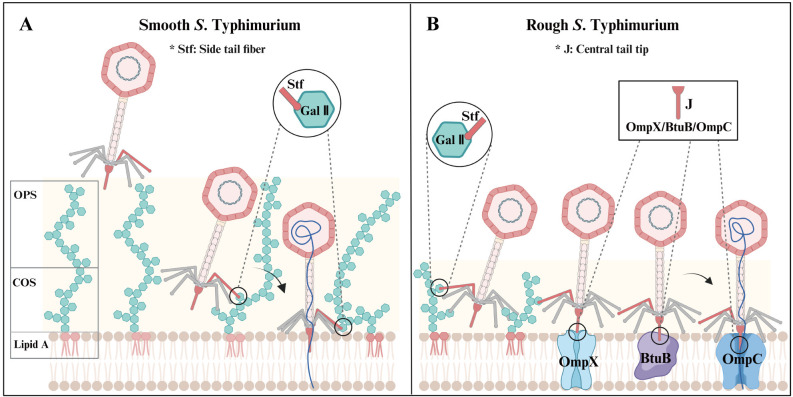
Two receptor-targeting mechanisms of Gifsy-1 in smooth and rough S. Typhimurium strains. (A) The mechanism employed by Gifsy-1 infection of smooth S. Typhimurium. The OPS act as a barrier to prevent Gifsy-1 from binding the OMPs. To bypass the OPS shield, the Gifsy-1 recognizes and binds the Gal Ⅱ of COS via the side tail fiber Stf to adsorb and release its genomes into smooth bacteria. (B) The mechanism employed by the Gifsy-1 infection of rough S. Typhimurium. Loss of OPS results in exposure of the OM components to the phage. The Gifsy-1 adsorbs on the rough strain by Stf, which targeted the Gal Ⅱ moiety of COS and by the central tail tip J, which interacts with three OMPs: OmpX, BtuB, and OmpC. After the J protein irreversibly binds OmpC, the phage ejects its genome into rough bacteria. This figure was created with Biorender.

This study elucidated the sophisticated receptor-targeting mechanisms employed by the phage Gifsy-1 to infect *S*. Typhimurium (Fig 10). The outermost OPS of the smooth strain constitute a barrier that impedes Gifsy-1 infection by shielding OMPs (Fig 10A). However, Gifsy-1 utilizs a strategy to bypass this barrier via its functional RBP, the side tail fiber Stf, to specifically recognize and bind the necessary receptor, the Gal Ⅱ moiety of COS, facilitating initial adsorption and terminal DNA ejection (Fig 10A). Loss of OPS leads to increased exposure of the OM components of the host to the phage (Fig 10B). Gifsy-1 employs a four-receptor targeting mode for host infection, enhancing its lytic activity on rough *S*. Typhimurium. Gifsy-1 adsorbs onto the rough strain via tail fibers Stf-mediated recognition of Gal Ⅱ and J-mediated binding of three OMPs: OmpX, BtuB, and OmpC (Fig 10B). The ejection of DNA into the rough strain is triggered by the J protein binding OmpC irreversibly (Fig 10B). These two receptor-targeting mechanisms enable Gifsy-1 to adapt to smooth or rough LPS variations in *S*. Typhimurium.

## Discussion

Characterizing phage‒host interactions is important for understanding their ecological functions and developing rational phage therapies. Only a minority of available phages have been studied in terms of their receptor targeting abilities [14]. One of the major reasons is that the characterization of phage receptors is time-consuming and challenging. The performance of λ and λ-like siphophages has been extensively investigated in rough bacteria rather than smooth bacteria [18]. In this study, we revealed the receptor-targeting mechanism by which the temperate phage Gifsy-1 exploits multiple surface receptors to adsorb on and eject DNA into both smooth and rough *S*. Typhimurium. Impressively, Gifsy-1 adopts two targeting strategies according to the status of OPS expression in *S*. Typhimurium. Interactions focused mainly on the central tail tip J-OmpC and the side tail fiber Stf-COS Gal Ⅱ are alternatively exploited by Gifsy-1 to infect rough and smooth strains, respectively.

Gifsy-1 was previously found to be less efficient at infecting smooth *S*. Typhimurium strains because of the presence of the outer core and OPS [37]. Our study further demonstrated that the outer core is irrelevant; rather bacterial resistance to Gifsy-1 phage is mediated by OPS. Similar phenomena have also been observed in other temperate phages, including the coliphage λ [18], which is blocked by the O16 OPS of *E. coli*; the Stx-converting phage phi24B [38], whose lysogenization of non-O157 *E. coli* strains is effectively restricted by OPS; and a series of lytic *Tevenvirinae* phages, such as the T2, T4, and T6 coliphages [18]*.* In contrast, strikingly, Gifsy-1 can infect multiple smooth *S. enterica* strains that produce different OPS types, suggesting that Gifsy-1 possesses specific mechanisms to penetrate various OPS layers. *S. enterica* strains from different serogroups exhibit diverse susceptibility to Gifsy-1. The bacteria in serogroup C1 are all resistant to Gifsy-1 infection, which is in sharp contrast to the high susceptibility of *S.* Typhimurium or *S.* Enteritidis. OPS structures determine the classification of serogroups. Among the five tested serogroups, C1 OPS possesses the longest main chain with pentasaccharide O units, whereas B and D1 share identical and the shortest main chain but differ in side-branch residues [16]. Structural heterogeneity may underlie the variable barrier function of OPS against Gifsy-1.

Most gram-negative bacteria contain gene clusters for the synthesis of OPS [33]. To promote propagation, phages have evolved to infect smooth strains via recognition of OPS as a primary receptor. The P22-like *Shigella* phage Sf6 reversibly binds the OPS of the *Shigella* Y serotype via enzymatically active tail spikes, which is an essential step to allow the phage further reach and contact with the secondary receptors OmpA and OmpC [39]. Additionally, the coliphage T5 can infect smooth *E. coli* by successive recognition of the OPS via the side fiber pb1 and OMP FhuA via the central tail fiber pb5 [18]. However, the drawback of this strategy is that bacteria are completely resistant to phages once the OPS is lost (P22 and Sf6) or converted (T5-like DT57C) [39–41]. Thus, the host ranges of these phages exclusively rely on speciﬁc recognition of the OPS. In the smooth strain of *S*. Typhimurium, OPS abolished the binding of Gifsy-1 to OmpC, which is essential for infecting the rough strain. Instead, Gifsy-1 exploits the side tail fiber Stf to target Gal Ⅱ of COS as the sole receptor, allowing it to adsorb and eject DNA into smooth strains. This COS-targeting feature has not been identified in λ-like siphophages, but is similar to the infection modes of the podophage T7 and the myophages P1 and P2. These phages are considered to target COS as the terminal receptor; however, the specific recognition site on the LPS core has yet to be determined. Moreover, the loss of host susceptibility to these phages correlates with the surface display of OPS, which covers the COS receptor upon phage adsorption [18]. Thus, although the COS is physically closer to the OPS than OMPs, phages must require a strategy to penetrate the OPS to access the COS.

Other surface polysaccharides are also utilized by phages to bypass the OPS barrier through elusive mechanisms. Enterobacterial common antigen (ECA), adjacent to LPS at the OM, is targeted by the *Myoviridae* phage phi92 and the *Podoviridae* phage N4 as the primary receptor [18]. Phage N4 also recognizes another polysaccharide primary receptor, the N4 glycan receptor (NGR), promoting subsequent binding to the terminal receptor NfrA (OM porin) [42]. In contrast to OPS, the variability of COS, ECA, and NGR is notably limited, efficiently providing phages with reliable targets for multiple hosts. Consistent with the bactericidal properties of phi92 and N4 [18,43], Gifsy-1 also has a broad host range and can infect both smooth and rough bacterial hosts. This feature enables these phages to be promising and priority choices for phage therapy.

Two types of LPS are present in natural bacterial environments; for instance, rough LPS is predominant in *Burkholderia cepacia* during chronic infection [44]. In *S. enterica*, OPS production is regulated according to the growth phase by RpoN/S and increases mainly in the stationary phase [45,46]. Moreover, the phase variation in *Salmonella* LPS is regulated by the *opvAB* (OPS phase variation) operon, allowing bacteria to switch between smooth LPS (OpvAB^OFF^) and rough LPS (OpvAB^ON^) [47]. Smooth LPS, which is prevalent *in vivo*, enhances bacterial virulence and immune evasion; however, after macrophage invasion the OPS is shortened for better bacterial diffusion and cell migration in the host [48]. Rough LPS, which is favoured in OPS-targeted phage-filled environments, provides phage resistance [49]. This reversible phase variation allows *S. enterica* to adapt swiftly to different threats, switching LPS types as needed for survival in host or external settings. The antimicrobial strategies for rough strains are as important as those for smooth strains. Regardless of the receptor-targeting strategies used, versatile phages ultimately employ mechanical forces, arising from tail protein conformation changes, to pass through the OPS layer [17]. Therefore, exploring the molecular basis that induces conformational changes in COS-targeting Gifsy-1 or other conserved carbohydrate-targeting phages to penetrate the OPS layer would be valuable in future studies.

OmpC is a known receptor for Gifsy-1 infection of rough *S.* Typhimurium strains [27]. Here, we verified this conclusion and further identified three other primary receptors, namely, Gal Ⅱ of COS and OM proteins (BtuB and OmpX), associated with this process. Exploitation of multiple receptors for adsorption has also been observed in other phages, such as the *Myoviridae* phage Bp7, which has two primary receptors (LamB and OmpC) and one secondary receptor (COS) [32]. It is believed to be beneficial for phages to move and find an optimal position for irreversible binding [14]. Nevertheless, the three identified receptors were dispensable for the lytic activity of Gifsy-1. The loss of any one of these receptors only slightly affects the EOP. The high prevalence of OmpC on the surface of gram-negative bacteria [50,51], likely accounts for its frequent targeting by phages. Recognition of primary receptors has been shown to be optional for many siphophages [52]. This unique feature enables these phages to infect hosts through alternative pathways, highlighting their adaptable infection strategies. In contrast, binding the primary receptor is essential for interaction with the secondary receptor by many podoviruses [17]. This is why phage P22 is incapable of infecting rough *S. enterica*. OM receptors are targeted by the RBP-central tail tip J of Gifsy-1, which is expected and consistent with the mechanism in λ phage, which also uses the J protein to interact with the LamB receptor [35].

Interestingly, the two infection mechanisms of Gifsy-1 also demonstrated the dual role of the Gal Ⅱ of COS during phage‒host interactions, functioning as one of the primary receptors for infecting rough bacteria but as the sole receptor for smooth bacteria. This branched Gal Ⅱ was recognized by the side tail fiber Stf, indicating saccharide-binding domains within the tail protein. This finding was consistent with previous studies on other siphophages in which host polysaccharide receptors are targeted by side tail fibers [53]. The irreversible binding of secondary receptors rather than the reversible binding of primary receptors by phage proteins results in the ejection of DNA into the host cytoplasm [14]. Therefore, it could be inferred that Stf bound the same receptor, while differences in their binding affinities caused distinct outcomes, with or without genome release. Obviously, whether the OPS was displayed determined this discrepancy. We predicted that OPS could provide a stimulatory signal to induce a conformational change in the Stf that transforms the reversible binding of COS to irreversible binding; however, the molecular mechanisms underlying signal reception by specific proteins and the subsequent transduction processes remain intriguing and require further investigation.

## Materials and methods

### Bacterial strains and growth conditions

The bacterial strains and plasmids used in this study are listed in S2 and S3 Tables. The *S.* Typhimurium strain ATCC14028 (NZ_CP034479.1), which possesses multiple prophages [54], was used as the producer of Gifsy-1 and as the parent strain to construct the mutants SLT40 (ΔGifsy-3) and SLT41 (ΔGifsy-1ΔGifsy-3). A total of 171 clinical isolates of *S. enterica* [55] stored in our laboratory were used to determine the host range of Gifsy-1 and were listed in S1 Table. The *S*. Typhimurium strain S-13 (RCAD-S-097, GCA_026131865.1) is a non-lysogen and exhibits moderately susceptible to Gifsy-1. S-13 was used as an indicator for studying receptor-targeting mechanism of Gifsy-1. The bacterial strains were routinely grown at 37°C on solid or liquid Luria–Bertani (LB) medium, except for the *E. coli* BL21(DE3) strains harbouring recombinant pET28a or pCold plasmids, which were grown at 25°C or at 16°C when incubated with IPTG. The appropriate antibiotics were supplemented at the following concentrations: kanamycin, 50 μg/ml; chloramphenicol, 25 μg/ml; and ampicillin, 100 μg/ml.

### Construction of *S*. Typhimurium mutants and complemented strains

All the primers used in this study are listed in S4 Table. The genes of the ATCC14028 and S-13 strains were deleted via allelic exchange using the suicide plasmid pRE112, as described previously [56]. Briefly, for deletion of the gene cluster *RS13550-RS13880*, which encodes for elements synthesis of the prophage Gifsy-3 in ATCC14028, the upstream and downstream homologous arms were amplified by the primers D*RS13550-RS13880*-1F/1R and D*RS13550-RS13880*-2F/2R, respectively and then joined by PCR with the primers D*RS13550-RS13880*-1F/2R. The fragment was inserted into the pRE112 plasmid using a ready-to-use seamless cloning kit (Sangon Biotech, Chengdu, China). The generated plasmid was transformed into the ATCC14028 strain via conjugation with *E. coli* SM10 λ pir [57]. The mutant was selected via sucrose-based counterselection and confirmed via PCR with the primers D*RS13550-RS13880*-1F/2R. Other gene deletions were performed using the same procedure. For complementation, pET28a was used as the vector for expression of genes such as *ompX*, *btuB*, and *ompC*. The gene sequences were amplified by C*ompX*-F/R, C*btuB*-F/R, and C*ompC*-F/R and inserted into pET28a via a ready-to-use seamless cloning kit (Sangon Biotech). The recombinant plasmids were transformed into the corresponding strains via electroporation.

### Phage isolation and propagation

Prophages from the producer strain SLT40 or SLT41 were induced with hydrogen peroxide [58]. Briefly, an overnight bacterial culture was diluted and grown to the exponential phase (OD_600_ = 0.8), and hydrogen peroxide was added to a final concentration of 4 mM. After incubation for 3 h at 37°C, the phage suspensions were harvested through centrifugation at 12,000 rpm and 4°C for 10 min and then gently filtered through a sterile 0.45 μm membrane filter (Millipore, Darmstadt, Germany). The titre of the phage mixture was determined in a double-layer plaque assay (as described below). To propagate the phage Gifsy-1, as described previously [59], a single plaque from the ΔOPS-S-13 lysate plate was carefully selected using a sterile pipette tip, transferred to 500 μL of SM buffer (100 mM NaCl, 8 mM MgSO_4_·7H_2_O, 50 mM Tris-HCl; pH 7.5), and then vortexed to ensure complete resuspension of the phage. After centrifugation at 4,000 × g for 5 min to remove any remaining debris, the phage isolate was mixed with the indicator ΔOPS-S-13 and subjected to a double-layer plaque assay. The entire plate was lysed, and then 5 mL of SM buffer was poured on top of the plate with shaking at 90 rpm and incubated at room temperature for 15 min. The phage isolates from the top of the plate were collected, centrifuged, and filtered. The phage titre was determined before storage at 4°C.

### Double-layer plaque assay

The phage titre and EOP were determined in a double-layer plaque assay [60]. 100 µL of each appropriately diluted phage isolate was mixed with 50 µL of overnight culture of the indicator strain, which was then added to 5 mL of warm molten soft LB agar. This mixture was poured onto a solid agar plate and incubated at 37°C for 10 h, after which the phage titre was quantified as plaque-forming units (PFUs) per mL. The EOP was assessed via the following steps: 10 µL of serial dilutions of phage solution were spotted onto indicator strain lawns that were grown on top of soft agar. Clear plaque zones were monitored and photographed.

To determine the host range of Gifsy-1, 50 μL of overnight bacterial culture and 50 μL of phage solution from SLT40 or SLT41 were added to 5 mL of warm molten soft agar. The mixture was then poured over a solidified agar plate. The plates were incubated at 37°C overnight or until plaques developed. The susceptibility of bacteria to Gifsy-1 was determined by plaque formation induced by Gifsy-1 phage from the SLT40 strain, in contrast to the lack of plaque formation by phages from the SLT41 strain.

### Measurement of bacterial growth curves

Overnight cultures (10^9^ CFU/mL) of the indicator strains S-13, ΔOPS-S-13, Δ*waaG*-S-13, or Δ*waaC*-S-13 were diluted 1000-fold into 50 mL of fresh LB medium. Gifsy-1 (10^9^ PFU/mL) isolated from SLT40 was added to the culture at a multiplicity of infection (MOI) of 0.01. Additionally, the indicator was incubated with phage solution (without Gifsy-1) from SLT41 or cultured alone as a control. The culture was incubated at 37°C with shaking at 180 rpm, and the absorbance at 600 nm was measured hourly for 20 h.

### Phage adsorption assay

To ascertain the adsorption kinetics of Gifsy-1 on strains S-13, Δ*ompC*-S-13, ΔOPS-S-13, and Δ*ompC*ΔOPS-S-13, 200 µL of each strain (10^9^ CFU/mL) was mixed with an equal volume of phage (10^7^ PFU/mL) and incubated at 37°C for 20 min. At 5-min intervals, the samples were centrifuged at 4°C and 12,000 rpm for 2 min to pellet the bacteria, and the supernatant was collected to quantify the residual phage titre by double-layer plaque assay. A control was established by incubating bacterium-free LB medium with the phage to determine the total phage titre. Relative adsorption rate = [1-(residual phage titre/total phage titre)] ×100%.

### LPS extraction, protein expression and purification

LPS from S-13, derivative mutants, and complemented strains was extracted using an LPS extraction kit (iNtRON Biotechnology DR, Kyonggi-do, South Korea). The purity of the LPS was determined with a BCA Protein Assay Kit (Massachusetts, USA, Thermo Fisher Scientific) to assess protein contamination. The LPS phenotype was determined via SDS‒PAGE and silver staining as previously described [61].

For the expression of proteins including OmpC_-TF_, BtuB_-TF_, OmpX_-TF_, J_-TF_, GFP_-TF_, and GFP-J_-TF_, each gene sequence was fused with a His tag sequence, amplified and inserted into the pCold plasmid (TaKaRa, Tokyo, Japan). For expression of the Stf_-His_ and J_-Flag_ proteins, each gene sequence was cloned and inserted into the plasmid pET28a. The generated recombinant plasmids were subsequently transformed into *E. coli* BL21(DE3). The bacteria were then grown with 0.5 mM IPTG at 16°C or 25°C for 20 h; after which the protein was purified with Ni- nitrilotriacetic acid (NTA) beads (Smart Life Sciences, Changzhou, China), as previously described [62]. The purified proteins from pCold contained the solubility-enhancing tag TF, therefore, the TF tag was also purified as a control in our experiments.

### Phage lysis and adsorption inhibition assays

To test the effect of LPS on phage lysis, 100 μl of the Gifsy-1 isolate (10^6^ PFU/mL) was preincubated with 10 mM LPS or the solution control (PBST) at 37°C for 30 min, after which it was added to a culture of the indicator strain ΔOPS-S-13 or S-13 at a MOI of 0.01. The mixture was incubated with shaking at 37°C for 5.5 h or 10 h. The numbers of ΔOPS-S-13 or S-13 cells were subsequently determined via serial dilution and plate counting. The strain cultured alone with an equal volume of SM buffer was also detected. To test the inhibitory effects of LPS or protein receptors (OmpC_-TF-His_, BtuB_-TF-His_, and OmpX_-TF-His_) on Gifsy-1 adsorption, a 10^9^ CFU/ml bacterial culture was incubated with Gifsy-1 (MOI = 0.01), which was preincubated with or without 10 mM LPS or 2 mg/mL protein at 37°C for 1 h. After 20 min of incubation at 37°C, the relative phage adsorption rate was measured.

To test the RBP role of Gifsy-1 tail fibers, 200 μL of a culture of *S*. Typhimurium strain S-13 or ΔOPS-S-13 (10^9^ CFU/mL) was preincubated with 1 mg/ml protein (J_-TF_, Stf, or TF) or protein solution control for 30 min at 37°C. Then, 200 μl of the Gifsy-1 isolate (10^7^ PFU/ml) was added to the mixture and incubated at 37°C for 20 min. The phage adsorption rates were measured.

### Detection of fluorescently labelled bacteria

To determine which OM receptor could interact with Gifsy-1 J, 500 μl of GFP-J or GFP (2 mg/mL) was incubated with 500 μl of overnight cultures of ΔOPS-S-13, S-13, Δ*ompX*ΔOPS-S-13, Δ*btuB*ΔOPS-S-13, Δ*ompC*ΔOPS-S-13, Δ*waaG*-S-13, or the complemented strains (10^9^ CFU/ml) for 15 min at 37°C. After incubation, the bacterial cells were collected (5,000 rpm, 5 min at 4°C) and washed twice with PBS. The cells were then resuspended in 500 μl of PBS. The fluorescence intensity of the labelled bacterial cells was measured with a Thermo Scientific Varioskan LUX Multimode Microplate Reader.

### Pull-down assay and western blotting analysis

To identify other membrane proteins targeted by Gifsy-1, purified J_-TF-His_ protein (1 mg/mL) was preincubated with 10 μg of rabbit anti-His_6_ monoclonal antibody (mAb) (Proteintech, Manchester, UK). Then, the lysed whole-cell proteins from the indicator strain S-13 and 100 μL of protein A/G magnetic beads (Smart Life Sciences) were added to perform a pull-down assay at 4°C overnight. The proteins bound by J_-TF-His_ were analysed by SDS‒PAGE, followed by peptide mass fingerprinting. Pull-down assays and western blot analysis were further performed to examine the interaction of RBP-J with OmpX, OmpC, or BtuB. Briefly, BtuB_-TF-His_ (116.8 kDa), OmpX_-TF-His_ (66.8k Da), OmpC_-TF-His_ (90.3 kDa), and TF_-His_ (48.2 kDa) were purified and incubated with the J_-Flag_ protein (171.1 kDa) in BL21 (DE3) (*gpJ*-flag-pET28a) cell lysate at 4°C overnight. Then, the Ni-NTA beads were added and incubated for 2 h at 4°C. The proteins bound by the beads were washed and separated by SDS-PAGE. The specific protein bands were analysed by western blotting with a rabbit anti-His_6_ mAb (Proteintech) and a mouse anti-Flag mAb (MBL, Beijing, China).

### BLI assay

Purified His_6_-tag-fused proteins (OmpX_-TF_, OmpC_-TF_, BtuB_-TF_, and Stf_-TF_, 1 mg/ml) were incubated with rabbit anti-His mAb (Proteintech, 1:200) overnight at 4°C on a rotating wheel. Crude cell lysates of the J_-flag_ protein or purified LPS from the S-13, Δ*waaB*-S-13, and C*waaB*-Δ*waaB*-S-13 strains were prepared for the BLI assay. The interactions of OmpX-J, OmpC-J, BtuB-J, and Stf-LPS were detected in BLI assays using an Octet R2 instrument from SARTORIUS. PBS with 0.1% BSA and 0.01% Tween-20 was used as the assay buffer. The proteins bound with the anti-His mAb in advance were tethered to ProA biosensors (SARTORIUS, Gottingen, Germany) by dipping sensors into each protein mixture. Average saturation response levels of 5 nm were achieved in 13 min for all proteins. Sensors with tethered proteins were washed in assay buffer for 1 min to eliminate nonspecifically bound proteins and establish stable baselines before starting association‒dissociation cycles with different concentrations of test samples (J_-flag_ protein or LPS). The raw kinetic data collected were processed in Octet Analysis Studio 13.0 software via double reference subtraction, in which both the assay buffer reference and sensor tethered with the TF protein reference were subtracted. The resulting data were analyzed on the basis of a 1:1 binding model from which the association rate constant (*K*_on_) and dissociation rate constant (*K*_off_) values were obtained, and then the equilibrium dissociation constant (*K*_d_) values were calculated.

### Data analysis

The data are presented as the means ± standard deviations from a minimum of three independent biological replicates. Statistical comparisons were carried out via paired *t* tests followed by two-tailed calculations, one-way ANOVA or two-way ANOVA followed by Tukey’s multiple comparison test in GraphPad Prism software. A probability value of *p* < 0.05 was considered statistically signiﬁcant. The *in vitro* experiments were independently conducted three times.

## Supporting information

S1 Fig Analysis of LPS phenotypes by silver staining. LPS from the WT strain S-13, the OPS-deficient mutant ΔOPS-S-13, a series of COS mutants, and related complemented strains were extracted and analyzed by SDS‒PAGE and subsequent silver staining.
(TIF)

S2 Fig Identification of the COS moiety exploited by the Gifsy-1 via phage lysis inhibition assays by LPS. Gifsy-1 was incubated with LPS extracted from the WT S-13 strain, ΔOPS-S-13, Δ*waaI*Δ*waaB*-S-13, Δ*waaB*-S-13 or the related complemented strains. Then, phage lysis on ΔOPS-S-13 (A) or S-13 (B) were measured. Differences between groups were analyzed by one-way ANOVA followed by Tukey’s multiple comparison test. The asterisk above the error bar indicates a difference compared with the blank control group. ***, *p* < 0.001.
(TIF)

S3 Fig Sequence alignments of tail fibers J and Stf of Gifsy-1 andλ or Gifsy-1 and Ur-λ. (A) Amino acid sequence alignments of the J protein from λ phage (GenBank: NP_040600.1) and Gifsy-1 (WP_138930548.1). (B) Amino acid sequence alignments of the Stf proteins of Ur-λ (P03764.2) and Gifsy-1 (WP_001144691.1). Conserved residues, highlighted by a blue background to denote at least 30% sequence identity, were predominant within the N-terminal domain (approximately 964 amino acids) of the J protein, whereas they were dispersed throughout the domains of Stf.
(TIF)

S4 Fig Identification of protein receptors involved in Gifsy-1 infection of rough *S.* Typhimurium strains. The phage lysis (A), adsorption (B) and EOP (C) of the Gifsy-1 were measured on the ΔOPS-S-13 and its derived mutants, and some complemented strains. Differences between groups were analyzed by one-way ANOVA followed by Tukey’s multiple comparison test. The asterisk above the error bar indicates a difference compared with the ΔOPS-S-13 group. The asterisk above the line indicates a signiﬁcant difference between the two indicated groups. ***, *p* < 0.001.
(TIF)

S5 Fig Determination of receptors targeted by Gifsy-1 J protein via fluorescence labelling. The bacterial strains including ΔOPS-S-13, S-13, various mutants, and complemented strains were incubated with the GFP-J fusion protein or GFP, respectively. The fluorescence intensity labeled on bacteria was subsequently detected. The differences between groups were analyzed by two-way ANOVA followed by the Tukey multiple comparison test. The asterisk above the line indicates a signiﬁcant difference between the two indicated groups. ***, *p <* 0.001.
(TIF)

S6 Fig Detection of the interaction of the solubility-enhancing tag protein TF_-His_ and Gifsy-1 J_-Flag_. The tag TF was expressed and purified from BL21(DE3) harboring pCold. Then the interaction of TF_-His_ and J_-Flag_ was analyzed by pull-down assay and western blotting with anti-His mAb and anti-Flag mAb.
(TIF)

S1 Table List of bacterial strains used in this study. (XLSX)

S2 Table List of plasmids used in this study. (XLSX)

S3 Table Susceptibility of 171 clinical strains to Gifsy-1 infection. “+” indicated positive susceptible to Gifsy-1; “-” indicated resistant to Gifsy-1.
(XLSX)

S4 Table List of oligonucleotide sequences of the primers used in this study. (XLSX)

S1 Data Raw data used to generate article figures. (ZIP)

## References

[1] Touchon M, Bernheim A, Rocha EP. Genetic and life-history traits associated with the distribution of prophages in bacteria. ISME J. 2016;10(11):2744–54. doi: 10.1038/ismej.2016.47 27015004 PMC5113838

[2] Kim M-S, Bae J-W. Lysogeny is prevalent and widely distributed in the murine gut microbiota. ISME J. 2018;12(4):1127–41. doi: 10.1038/s41396-018-0061-9 29416123 PMC5864201

[3] Chiang YN, Penadés JR, Chen J. Genetic transduction by phages and chromosomal islands: The new and noncanonical. PLoS Pathog. 2019;15(8):e1007878. doi: 10.1371/journal.ppat.1007878 31393945 PMC6687093

[4] Meyer JR, Dobias DT, Weitz JS, Barrick JE, Quick RT, Lenski RE. Repeatability and contingency in the evolution of a key innovation in phage lambda. Science. 2012;335(6067):428–32. doi: 10.1126/science.1214449 22282803 PMC3306806

[5] Brown EM, Arellano-Santoyo H, Temple ER, Costliow ZA, Pichaud M, Hall AB, et al. Gut microbiome ADP-ribosyltransferases are widespread phage-encoded fitness factors. Cell Host Microbe. 2021;29(9):1351-1365.e11. doi: 10.1016/j.chom.2021.07.011 34403684 PMC8429246

[6] Li D, Tang F, Xue F, Ren J, Liu Y, Yang D, et al. Prophage phiv142-3 enhances the colonization and resistance to environmental stresses of avian pathogenic Escherichia coli. Vet Microbiol. 2018;218:70–7. doi: 10.1016/j.vetmic.2018.03.017 29685224

[7] Gordillo Altamirano FL, Barr JJ. Phage Therapy in the Postantibiotic Era. Clin Microbiol Rev. 2019;32(2):e00066-18. doi: 10.1128/CMR.00066-18 30651225 PMC6431132

[8] Strathdee SA, Hatfull GF, Mutalik VK, Schooley RT. Phage therapy: From biological mechanisms to future directions. Cell. 2023;186(1):17–31. doi: 10.1016/j.cell.2022.11.017 36608652 PMC9827498

[9] Park JY, Moon BY, Park JW, Thornton JA, Park YH, Seo KS. Genetic engineering of a temperate phage-based delivery system for CRISPR/Cas9 antimicrobials against Staphylococcus aureus. Sci Rep. 2017;7:44929. doi: 10.1038/srep44929 28322317 PMC5359561

[10] Yosef I, Manor M, Kiro R, Qimron U. Temperate and lytic bacteriophages programmed to sensitize and kill antibiotic-resistant bacteria. Proc Natl Acad Sci U S A. 2015;112(23):7267–72. doi: 10.1073/pnas.1500107112 26060300 PMC4466736

[11] Burkal’tseva MV, Krylov SV, Kropinskiĭ AM, Pletneva EA, Shaburova OV, Krylov VN. Bacteriophage phi297--the new species of temperate phages Pseudomonas aeruginosa with a mosaic genome: potential use in phagotherapy. Genetika. 2011;47(7):900–4. 21938953

[12] Kilcher S, Studer P, Muessner C, Klumpp J, Loessner MJ. Cross-genus rebooting of custom-made, synthetic bacteriophage genomes in L-form bacteria. Proc Natl Acad Sci U S A. 2018;115(3):567–72. doi: 10.1073/pnas.1714658115 29298913 PMC5776983

[13] Wu M-Y, Chen L, Chen Q, Hu R, Xu X, Wang Y, et al. Engineered Phage with Aggregation-Induced Emission Photosensitizer in Cocktail Therapy against Sepsis. Adv Mater. 2023;35(6):e2208578. doi: 10.1002/adma.202208578 36440662

[14] Nobrega FL, Vlot M, de Jonge PA, Dreesens LL, Beaumont HJE, Lavigne R, et al. Targeting mechanisms of tailed bacteriophages. Nat Rev Microbiol. 2018;16(12):760–73. doi: 10.1038/s41579-018-0070-8 30104690

[15] Bertozzi Silva J, Storms Z, Sauvageau D. Host receptors for bacteriophage adsorption. FEMS Microbiol Lett. 2016;363(4):fnw002. doi: 10.1093/femsle/fnw002 26755501

[16] Liu B, Knirel YA, Feng L, Perepelov AV, Senchenkova SN, Reeves PR, et al. Structural diversity in Salmonella O antigens and its genetic basis. FEMS Microbiol Rev. 2014;38(1):56–89. doi: 10.1111/1574-6976.12034 23848592

[17] Letarov AV. Bacterial Virus Forcing of Bacterial O-Antigen Shields: Lessons from Coliphages. Int J Mol Sci. 2023;24(24):17390. doi: 10.3390/ijms242417390 38139217 PMC10743462

[18] Maffei E, Shaidullina A, Burkolter M, Heyer Y, Estermann F, Druelle V, et al. Systematic exploration of Escherichia coli phage-host interactions with the BASEL phage collection. PLoS Biol. 2021;19(11):e3001424. doi: 10.1371/journal.pbio.3001424 34784345 PMC8594841

[19] Kim M, Kim S, Park B, Ryu S. Core lipopolysaccharide-specific phage SSU5 as an Auxiliary Component of a Phage Cocktail for Salmonella biocontrol. Appl Environ Microbiol. 2014;80(3):1026–34. doi: 10.1128/AEM.03494-13 24271179 PMC3911222

[20] Prokhorov NS, Riccio C, Zdorovenko EL, Shneider MM, Browning C, Knirel YA, et al. Function of bacteriophage G7C esterase tailspike in host cell adsorption. Mol Microbiol. 2017;105(3):385–98. doi: 10.1111/mmi.13710 28513100

[21] Andres D, Hanke C, Baxa U, Seul A, Barbirz S, Seckler R. Tailspike interactions with lipopolysaccharide effect DNA ejection from phage P22 particles in vitro. J Biol Chem. 2010;285(47):36768–75. doi: 10.1074/jbc.M110.169003 20817910 PMC2978605

[22] Washizaki A, Yonesaki T, Otsuka Y. Characterization of the interactions between Escherichia coli receptors, LPS and OmpC, and bacteriophage T4 long tail fibers. Microbiologyopen. 2016;5(6):1003–15. doi: 10.1002/mbo3.384 27273222 PMC5221442

[23] Suga A, Kawaguchi M, Yonesaki T, Otsuka Y. Manipulating Interactions between T4 Phage Long Tail Fibers and Escherichia coli Receptors. Appl Environ Microbiol. 2021;87(13):e0042321. doi: 10.1128/AEM.00423-21 33893116 PMC8315975

[24] Steinbacher S, Miller S, Baxa U, Weintraub A, Seckler R. Interaction of Salmonella phage P22 with its O-antigen receptor studied by X-ray crystallography. Biol Chem. 1997;378(3–4):337–43. doi: 10.1515/bchm.1997.378.3-4.337 9165091

[25] Dhillon TS, Poon AP, Chan D, Clark AJ. General transducing phages like Salmonella phage P22 isolated using a smooth strain of Escherichia coli as host. FEMS Microbiol Lett. 1998;161(1):129–33. doi: 10.1111/j.1574-6968.1998.tb12938.x 9561740

[26] Chatterjee S, Rothenberg E. Interaction of bacteriophage l with its E. coli receptor, LamB. Viruses. 2012;4(11):3162–78. doi: 10.3390/v4113162 23202520 PMC3509688

[27] Ho TD, Slauch JM. OmpC is the receptor for Gifsy-1 and Gifsy-2 bacteriophages of Salmonella. J Bacteriol. 2001;183(4):1495–8. doi: 10.1128/JB.183.4.1495-1498.2001 11157969 PMC95030

[28] Andres D, Roske Y, Doering C, Heinemann U, Seckler R, Barbirz S. Tail morphology controls DNA release in two Salmonella phages with one lipopolysaccharide receptor recognition system. Mol Microbiol. 2012;83(6):1244–53. doi: 10.1111/j.1365-2958.2012.08006.x 22364412

[29] Kurasz JE, Crawford MC, Porwollik S, Gregory O, Tadlock KR, Balding EC, et al. Strain-Specific Gifsy-1 Prophage Genes Are Determinants for Expression of the RNA Repair Operon during the SOS Response in Salmonella enterica Serovar Typhimurium. J Bacteriol. 2023;205(1):e0026222. doi: 10.1128/jb.00262-22 36622230 PMC9879122

[30] Uppalapati S, Kant S, Liu L, Kim J-S, Orlicky D, McClelland M, et al. Prophage terminase with tRNase activity sensitizes Salmonella enterica to oxidative stress. Science. 2024;384(6691):100–5. doi: 10.1126/science.adl3222 38574144 PMC11443816

[31] Yates CR, Nguyen A, Liao J, Cheng RA. What’s on a prophage: analysis of Salmonella spp. prophages identifies a diverse range of cargo with multiple virulence- and metabolism-associated functions. mSphere. 2024;9(6):e0003124. doi: 10.1128/msphere.00031-24 38775467 PMC11332146

[32] Chen P, Sun H, Ren H, Liu W, Li G, Zhang C. LamB, OmpC, and the Core Lipopolysaccharide of Escherichia coli K-12 Function as Receptors of Bacteriophage Bp7. J Virol. 2020;94(12):e00325-20. doi: 10.1128/JVI.00325-20 32238583 PMC7307102

[33] Bertani B, Ruiz N. Function and Biogenesis of Lipopolysaccharides. EcoSal Plus. 2018;8(1):10.1128/ecosalplus.ESP-0001–2018. doi: 10.1128/ecosalplus.ESP-0001-2018 30066669 PMC6091223

[34] Werts C, Michel V, Hofnung M, Charbit A. Adsorption of bacteriophage lambda on the LamB protein of Escherichia coli K-12: point mutations in gene J of lambda responsible for extended host range. J Bacteriol. 1994;176(4):941–7. doi: 10.1128/jb.176.4.941-947.1994 8106335 PMC205142

[35] Gehring K, Charbit A, Brissaud E, Hofnung M. Bacteriophage lambda receptor site on the Escherichia coli K-12 LamB protein. J Bacteriol. 1987;169(5):2103–6. doi: 10.1128/jb.169.5.2103-2106.1987 2952637 PMC212104

[36] Guan J, Ibarra D, Zeng L. The role of side tail fibers during the infection cycle of phage lambda. Virology. 2019;527:57–63. doi: 10.1016/j.virol.2018.11.005 30463036 PMC6312755

[37] Ho TD, Slauch JM. Characterization of grvA, an antivirulence gene on the gifsy-2 phage in Salmonella enterica serovar typhimurium. J Bacteriol. 2001;183(2):611–20. doi: 10.1128/JB.183.2.611-620.2001 11133955 PMC94917

[38] Golomidova AK, Efimov AD, Kulikov EE, Kuznetsov AS, Belalov IS, Letarov AV. O antigen restricts lysogenization of non-O157 Escherichia coli strains by Stx-converting bacteriophage phi24B. Sci Rep. 2021;11(1):3035. doi: 10.1038/s41598-021-82422-x 33542282 PMC7862636

[39] Subramanian S, Dover JA, Parent KN, Doore SM. Host Range Expansion of Shigella Phage Sf6 Evolves through Point Mutations in the Tailspike. J Virol. 2022;96(16):e0092922. doi: 10.1128/jvi.00929-22 35894604 PMC9400499

[40] Golomidova AK, Kulikov EE, Prokhorov NS, Guerrero-Ferreira RC, Ksenzenko VN, Tarasyan KK, et al. Complete genome sequences of T5-related Escherichia coli bacteriophages DT57C and DT571/2 isolated from horse feces. Arch Virol. 2015;160(12):3133–7. doi: 10.1007/s00705-015-2582-0 26350770

[41] Bohm K, Porwollik S, Chu W, Dover JA, Gilcrease EB, Casjens SR, et al. Genes affecting progression of bacteriophage P22 infection in Salmonella identified by transposon and single gene deletion screens. Mol Microbiol. 2018;108(3):288–305. doi: 10.1111/mmi.13936 29470858 PMC5912970

[42] Junkermeier EH, Hengge R. A Novel Locally c-di-GMP-Controlled Exopolysaccharide Synthase Required for Bacteriophage N4 Infection of Escherichia coli. mBio. 2021;12(6):e0324921. doi: 10.1128/mbio.03249-21 34903052 PMC8669469

[43] Schwarzer D, Buettner FFR, Browning C, Nazarov S, Rabsch W, Bethe A, et al. A multivalent adsorption apparatus explains the broad host range of phage phi92: a comprehensive genomic and structural analysis. J Virol. 2012;86(19):10384–98. doi: 10.1128/JVI.00801-12 22787233 PMC3457257

[44] Hassan AA, Coutinho CP, Sá-Correia I. Burkholderia cepacia Complex Species Differ in the Frequency of Variation of the Lipopolysaccharide O-Antigen Expression During Cystic Fibrosis Chronic Respiratory Infection. Front Cell Infect Microbiol. 2019;9:273. doi: 10.3389/fcimb.2019.00273 31417878 PMC6686744

[45] Bravo D, Silva C, Carter JA, Hoare A, Álvarez SA, Blondel CJ, et al. Growth-phase regulation of lipopolysaccharide O-antigen chain length influences serum resistance in serovars of Salmonella. J Med Microbiol. 2008;57(Pt 8):938–46. doi: 10.1099/jmm.0.47848-0 18628492

[46] Bittner M, Saldías S, Altamirano F, Valvano MA, Contreras I. RpoS and RpoN are involved in the growth-dependent regulation of rfaH transcription and O antigen expression in Salmonella enterica serovar Typhi. Microb Pathog. 2004;36(1):19–24. doi: 10.1016/j.micpath.2003.08.003 14643636

[47] Cota I, Blanc-Potard AB, Casadesús J. STM2209-STM2208 (opvAB): a phase variation locus of Salmonella enterica involved in control of O-antigen chain length. PLoS One. 2012;7(5):e36863. doi: 10.1371/journal.pone.0036863 22606300 PMC3350482

[48] Lähteenmäki K, Kyllönen P, Partanen L, Korhonen TK. Antiprotease inactivation by Salmonella enterica released from infected macrophages. Cell Microbiol. 2005;7(4):529–38. doi: 10.1111/j.1462-5822.2004.00483.x 15760453

[49] Cota I, Sánchez-Romero MA, Hernández SB, Pucciarelli MG, García-Del Portillo F, Casadesús J. Epigenetic Control of Salmonella enterica O-Antigen Chain Length: A Tradeoff between Virulence and Bacteriophage Resistance. PLoS Genet. 2015;11(11):e1005667. doi: 10.1371/journal.pgen.1005667 26583926 PMC4652898

[50] Delcour AH. Structure and function of pore-forming beta-barrels from bacteria. J Mol Microbiol Biotechnol. 2002;4(1):1–10. 11763966

[51] Belousov MV, Kosolapova AO, Fayoud H, Sulatsky MI, Sulatskaya AI, Romanenko MN, et al. OmpC and OmpF Outer Membrane Proteins of Escherichia coli and Salmonella enterica Form Bona Fide Amyloids. Int J Mol Sci. 2023;24(21):15522. doi: 10.3390/ijms242115522 37958507 PMC10649029

[52] Golomidova AK, Kulikov EE, Prokhorov NS, Guerrero-Ferreira RС, Knirel YA, Kostryukova ES, et al. Branched Lateral Tail Fiber Organization in T5-Like Bacteriophages DT57C and DT571/2 is Revealed by Genetic and Functional Analysis. Viruses. 2016;8(1):26. doi: 10.3390/v8010026 26805872 PMC4728585

[53] Fokine A, Rossmann MG. Molecular architecture of tailed double-stranded DNA phages. Bacteriophage. 2014;4(1):e28281. doi: 10.4161/bact.28281 24616838 PMC3940491

[54] Sargen MR, Helaine S. A prophage competition element protects Salmonella from lysis. Cell Host Microbe. 2024;32(12):2063-2079.e8. doi: 10.1016/j.chom.2024.10.012 39515326 PMC11840918

[55] Guan Y, Li Y, Li J, Yang Z, Zhu D, Jia R, et al. Phenotypic and genotypic characterization of antimicrobial resistance profiles in Salmonella isolated from waterfowl in 2002-2005 and 2018-2020 in Sichuan, China. Front Microbiol. 2022;13:987613. doi: 10.3389/fmicb.2022.987613 36274743 PMC9582774

[56] Zhao X, Zeng X, Dai Q, Hou Y, Zhu D, Wang M, et al. Immunogenicity and protection efficacy of a Salmonella enterica serovar Typhimurium fnr, arcA and fliC mutant. Vaccine. 2021;39(3):588–95. doi: 10.1016/j.vaccine.2020.12.002 33341307

[57] Rubirés X, Saigi F, Piqué N, Climent N, Merino S, Albertí S, et al. A gene (wbbL) from Serratia marcescens N28b (O4) complements the rfb-50 mutation of Escherichia coli K-12 derivatives. J Bacteriol. 1997;179(23):7581–6. doi: 10.1128/jb.179.23.7581-7586.1997 9393727 PMC179713

[58] Frye JG, Porwollik S, Blackmer F, Cheng P, McClelland M. Host gene expression changes and DNA amplification during temperate phage induction. J Bacteriol. 2005;187(4):1485–92. doi: 10.1128/JB.187.4.1485-1492.2005 15687213 PMC545606

[59] Bonilla N, Rojas MI, Netto Flores Cruz G, Hung S-H, Rohwer F, Barr JJ. Phage on tap-a quick and efficient protocol for the preparation of bacteriophage laboratory stocks. PeerJ. 2016;4:e2261. doi: 10.7717/peerj.2261 27547567 PMC4975003

[60] Luong T, Salabarria A-C, Edwards RA, Roach DR. Standardized bacteriophage purification for personalized phage therapy. Nat Protoc. 2020;15(9):2867–90. doi: 10.1038/s41596-020-0346-0 32709990

[61] Hitchcock PJ, Brown TM. Morphological heterogeneity among Salmonella lipopolysaccharide chemotypes in silver-stained polyacrylamide gels. J Bacteriol. 1983;154(1):269–77. doi: 10.1128/jb.154.1.269-277.1983 6187729 PMC217456

[62] Zhao X, Wang W, Zeng X, Xu R, Yuan B, Yu W, et al. Klebicin E, a pore-forming bacteriocin of Klebsiella pneumoniae, exploits the porin OmpC and the Ton system for translocation. J Biol Chem. 2024;300(3):105694. doi: 10.1016/j.jbc.2024.105694 38301890 PMC10906532

