# Supplementary figures and images for: Two receptor-targeting mechanisms of lambda-like siphophage Gifsy-1 of Salmonella Typhimurium
Source: PLoS Pathog. 2025 Jul 31;21(7):e1013352. doi: 10.1371/journal.ppat.1013352 (PMC12312913; doi:10.1371/journal.ppat.1013352)

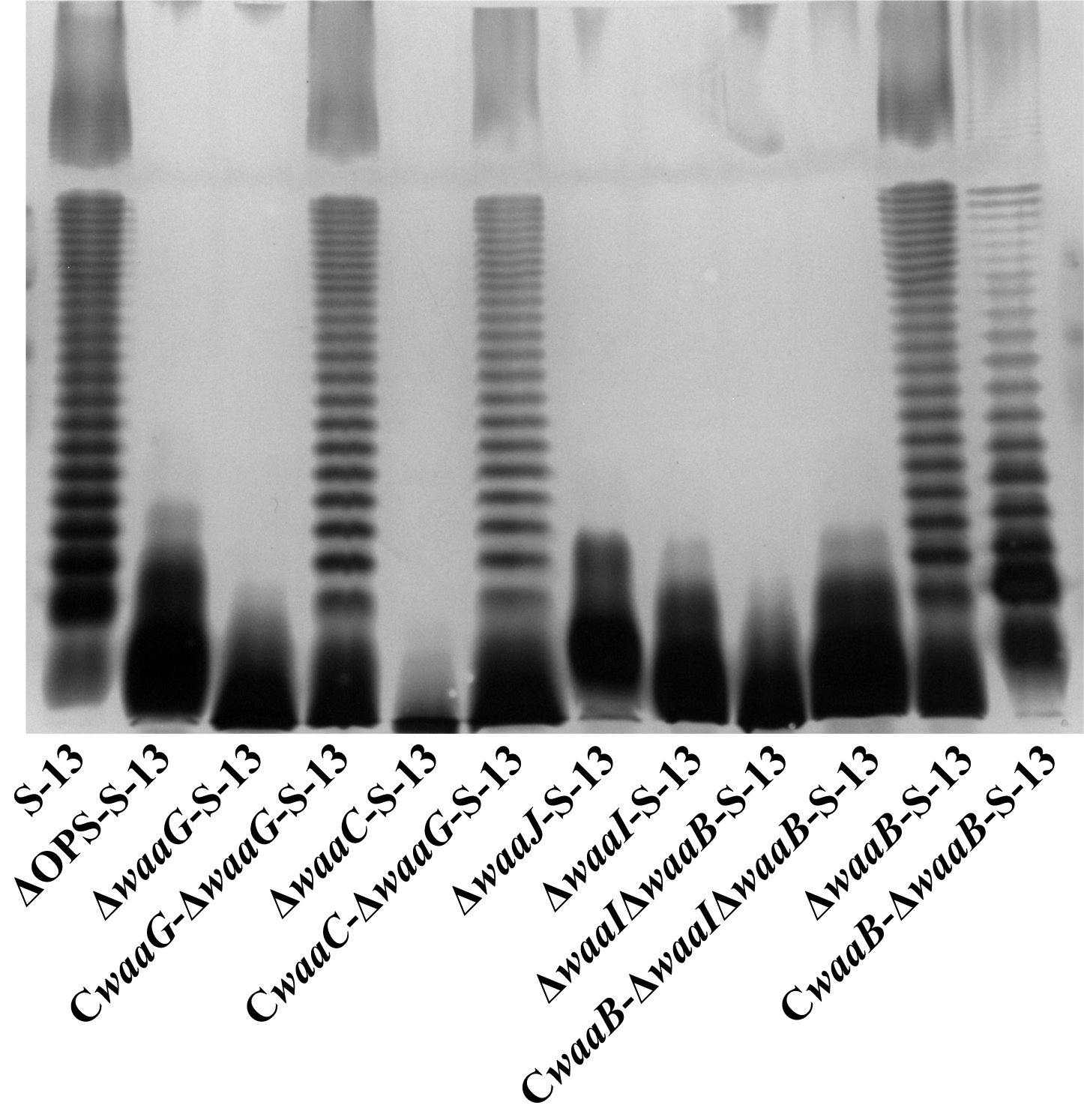

Supplement: S1 Fig — LPS from the WT strain S-13, the OPS-deficient mutant ΔOPS-S-13, a series of COS mutants, and related complemented strains were extracted and analyzed by SDS‒PAGE and subsequent silver staining. (TIF) [file ppat.1013352.s001.tif]

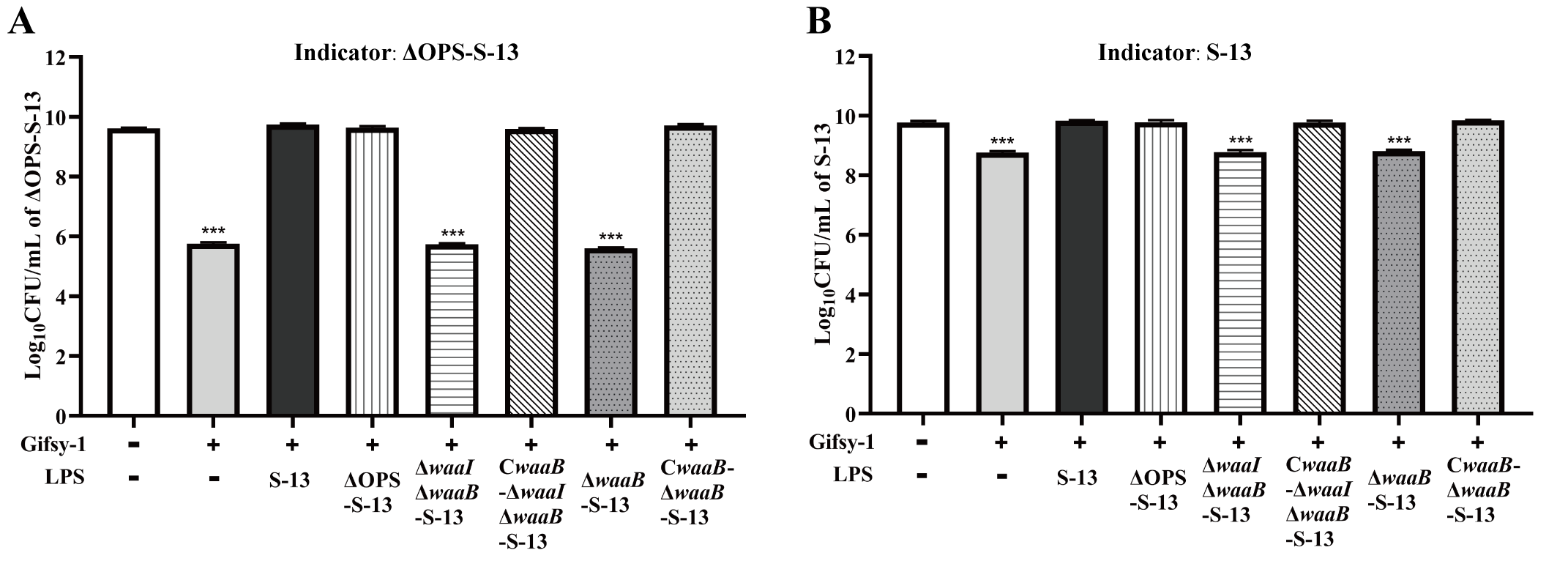

Supplement: S2 Fig — Gifsy-1 was incubated with LPS extracted from the WT S-13 strain, ΔOPS-S-13, ΔwaaIΔwaaB-S-13, ΔwaaB-S-13 or the related complemented strains. Then, phage lysis on ΔOPS-S-13 (A) or S-13 (B) were measured. Differences between groups were analyzed by one-way ANOVA followed by Tukey’s multiple comparison test. The asterisk above the error bar indicates a difference compared with the blank control group. ***, p < 0.001. (TIF) [file ppat.1013352.s002.tif]

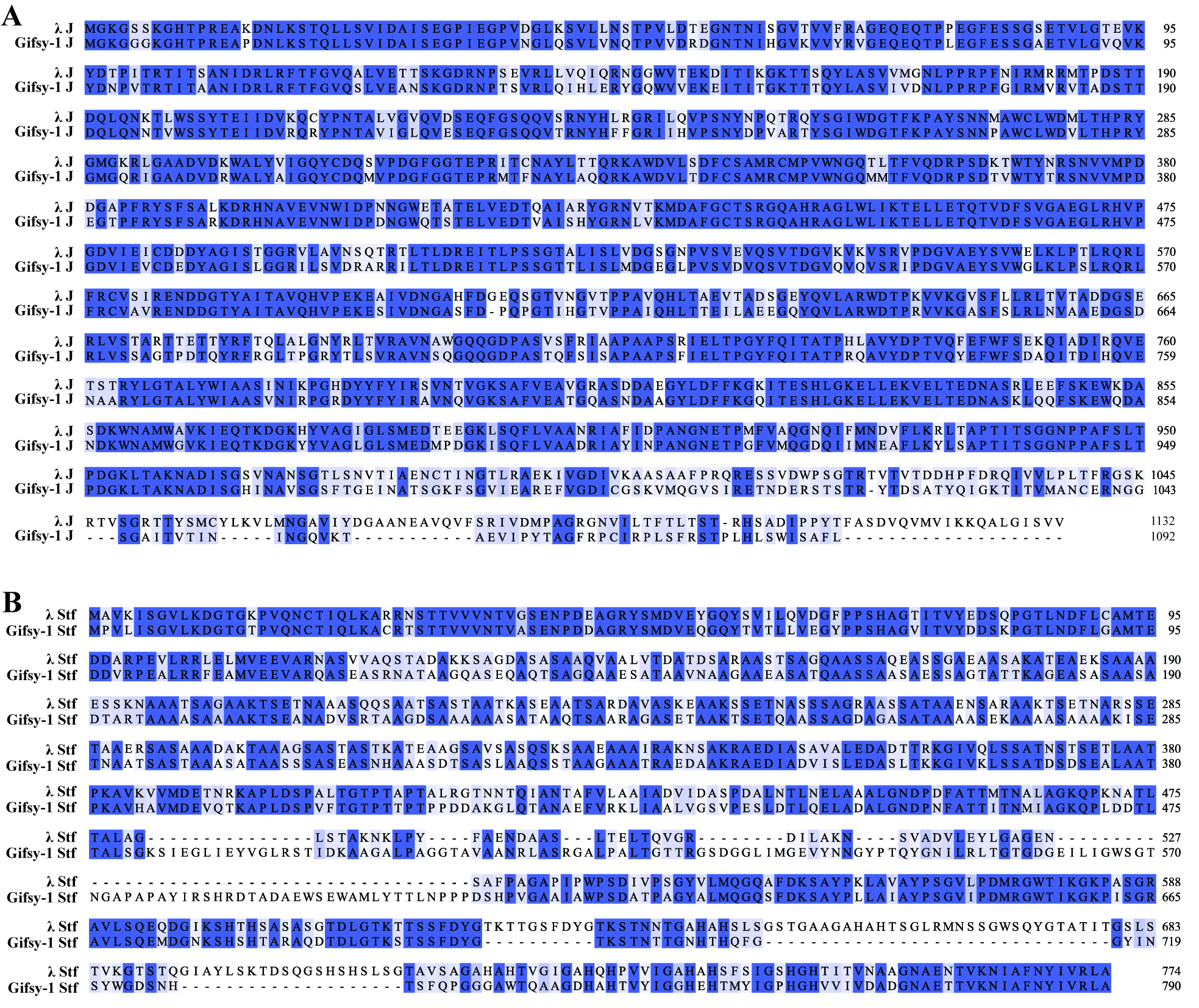

Supplement: S3 Fig — (A) Amino acid sequence alignments of the J protein from λ phage (GenBank: NP_040600.1) and Gifsy-1 (WP_138930548.1). (B) Amino acid sequence alignments of the Stf proteins of Ur-λ (P03764.2) and Gifsy-1 (WP_001144691.1). Conserved residues, highlighted by a blue background to denote at least 30% sequence identity, were predominant within the N-terminal domain (approximately 964 amino acids) of the J protein, whereas they were dispersed throughout the domains of Stf. (TIF) [file ppat.1013352.s003.tif]

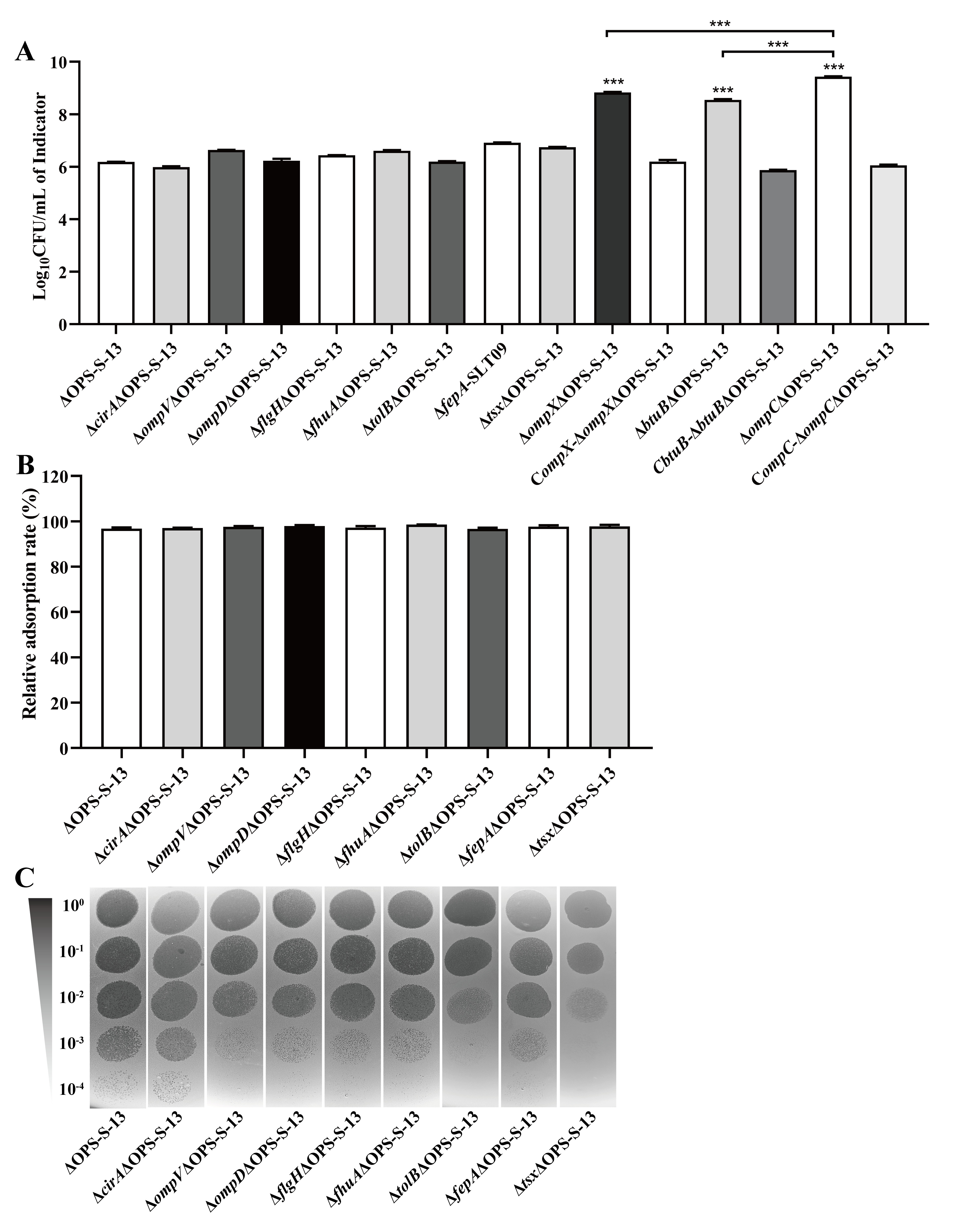

Supplement: S4 Fig — The phage lysis (A), adsorption (B) and EOP (C) of the Gifsy-1 were measured on the ΔOPS-S-13 and its derived mutants, and some complemented strains. Differences between groups were analyzed by one-way ANOVA followed by Tukey’s multiple comparison test. The asterisk above the error bar indicates a difference compared with the ΔOPS-S-13 group. The asterisk above the line indicates a signiﬁcant difference between the two indicated groups. ***, p < 0.001. (TIF) [file ppat.1013352.s004.tif]

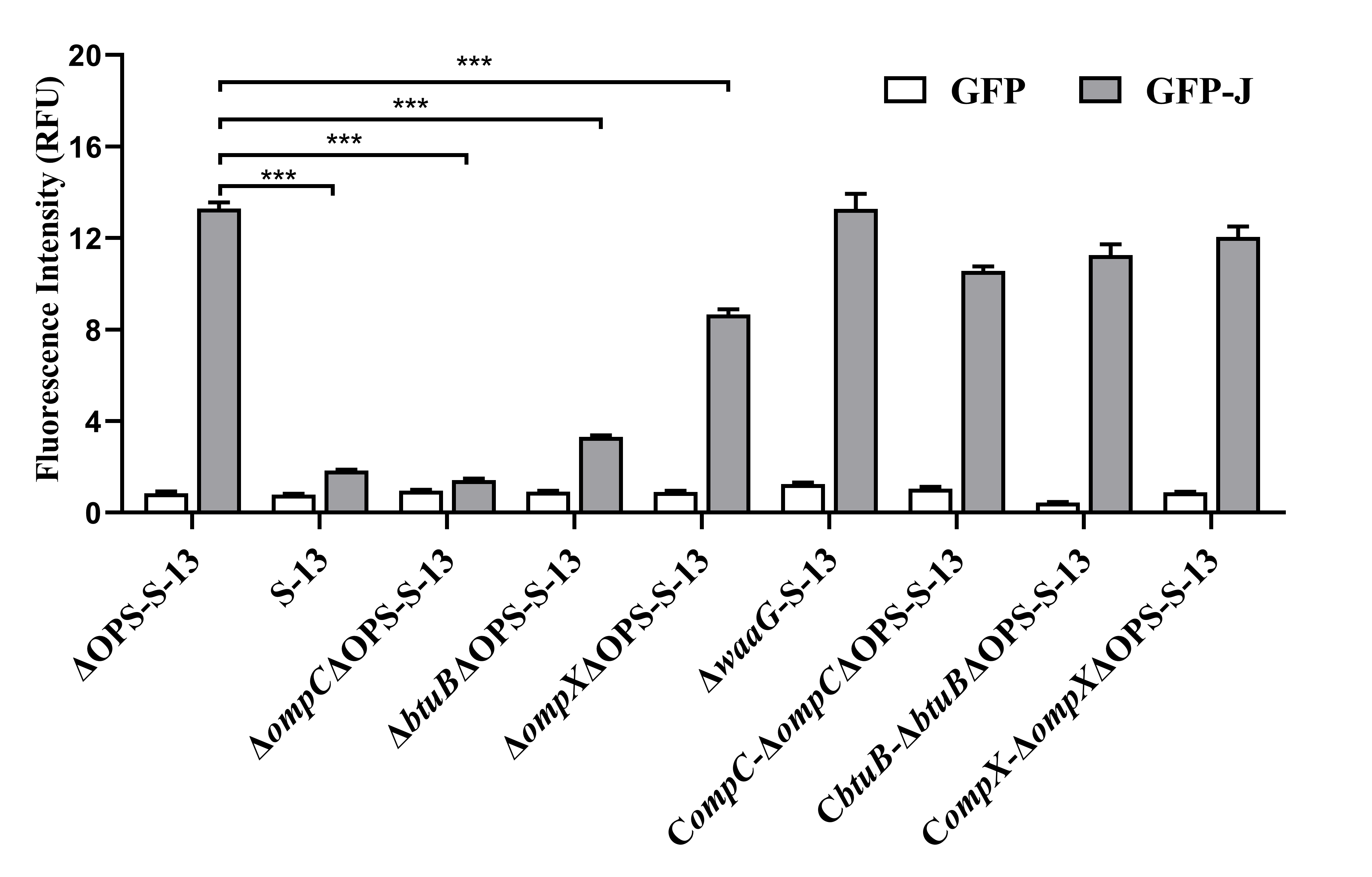

Supplement: S5 Fig — The bacterial strains including ΔOPS-S-13, S-13, various mutants, and complemented strains were incubated with the GFP-J fusion protein or GFP, respectively. The fluorescence intensity labeled on bacteria was subsequently detected. The differences between groups were analyzed by two-way ANOVA followed by the Tukey multiple comparison test. The asterisk above the line indicates a signiﬁcant difference between the two indicated groups. ***, p < 0.001. (TIF) [file ppat.1013352.s005.tif]

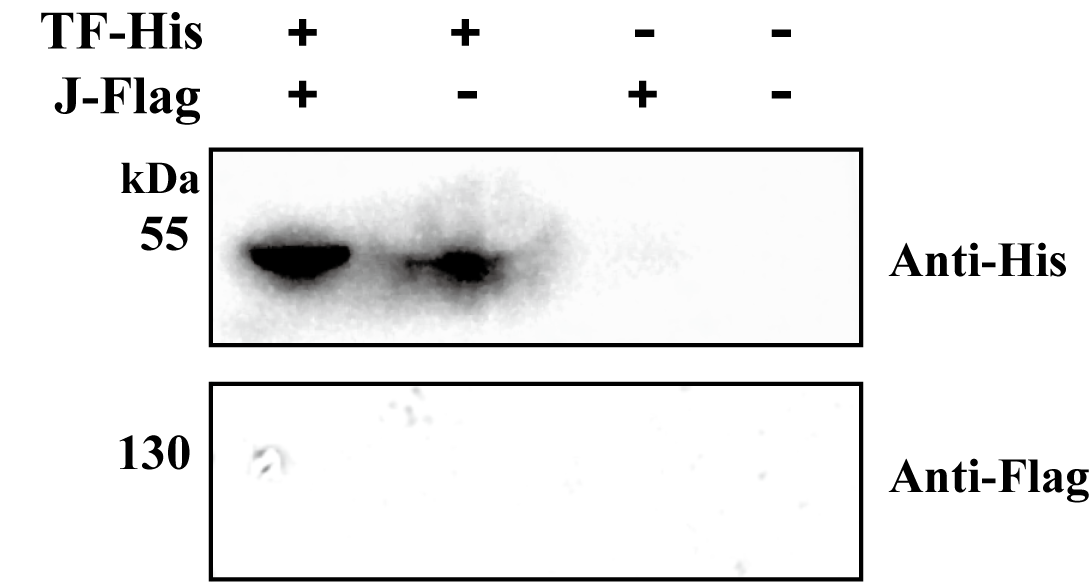

Supplement: S6 Fig — The tag TF was expressed and purified from BL21(DE3) harboring pCold. Then the interaction of TF-His and J-Flag was analyzed by pull-down assay and western blotting with anti-His mAb and anti-Flag mAb. (TIF) [file ppat.1013352.s006.tif]

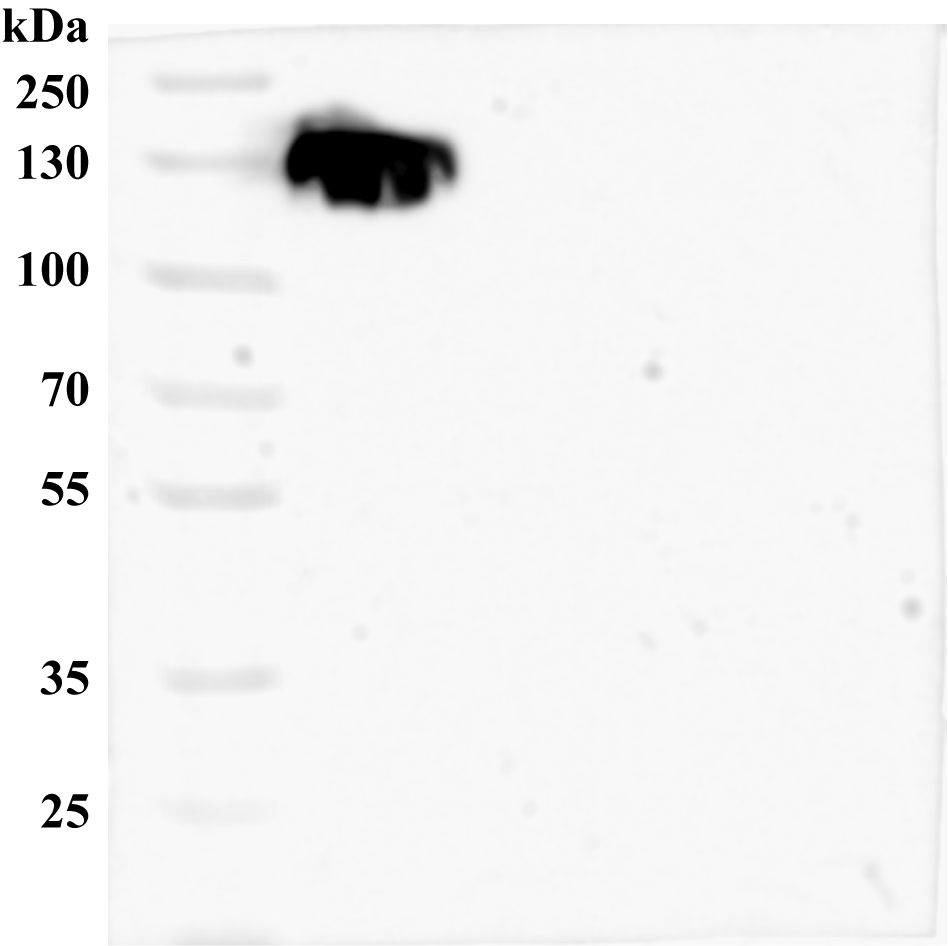

Supplement: S1 Data — (ZIP) [file ppat.1013352.s011.zip › All_raw_data/Raw_data_Fig_9A_Anti-Flag_OmpC-TF-His+J-Flag.tif]

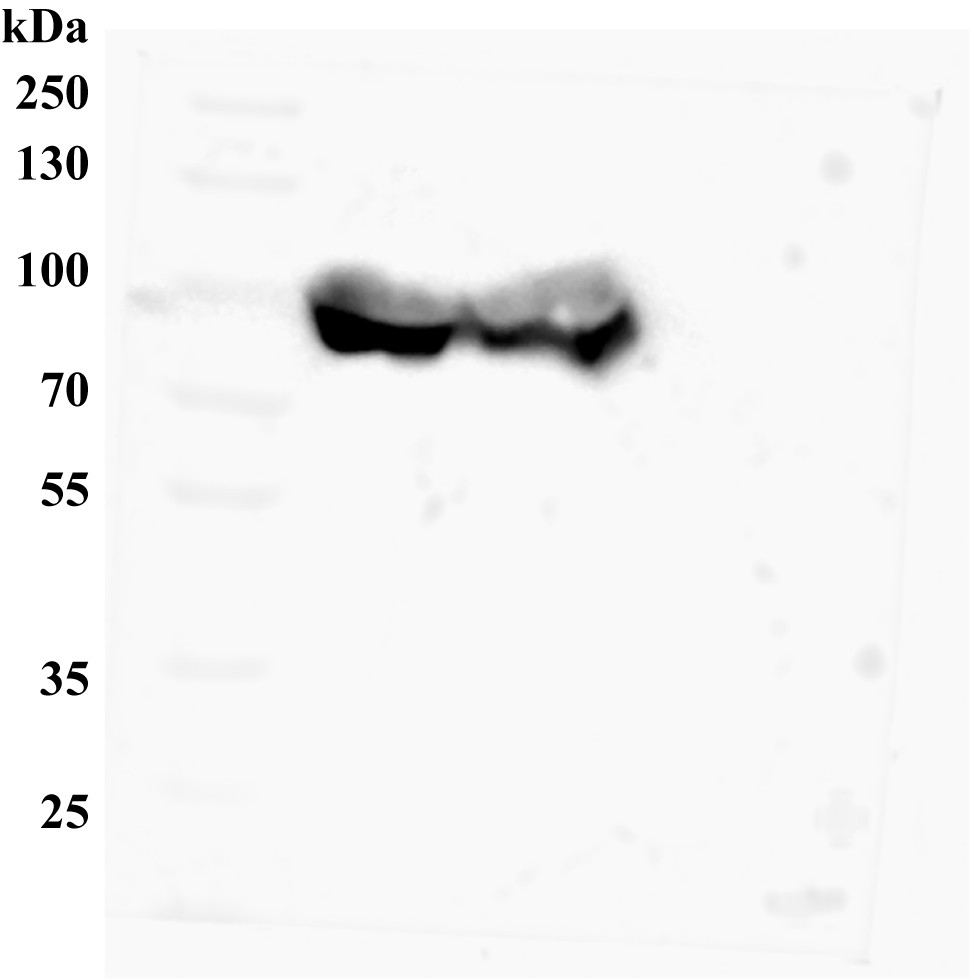

Supplement: S1 Data — (ZIP) [file ppat.1013352.s011.zip › All_raw_data/Raw_data_Fig_9A_Anti-His_OmpC-TF-His+J-Flag.tif]

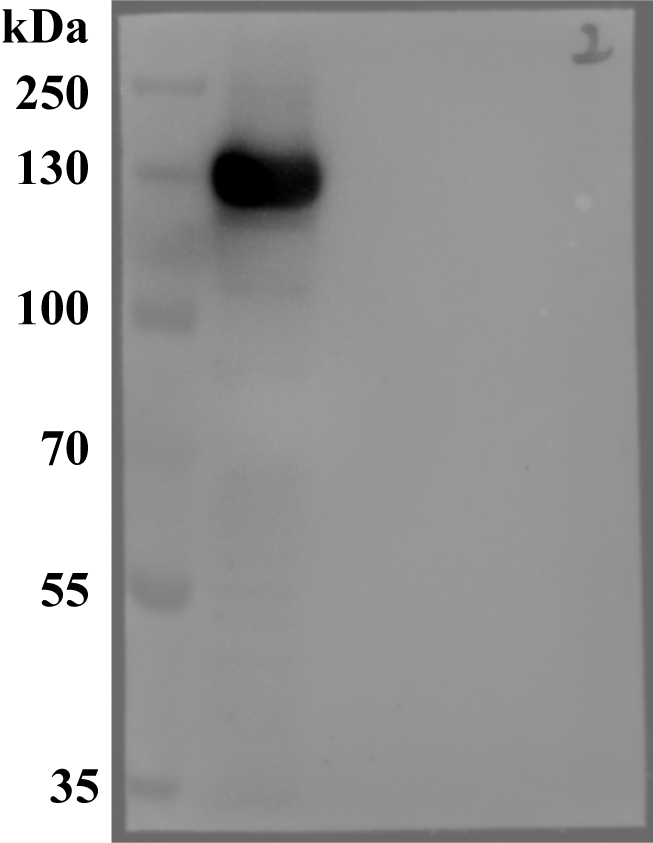

Supplement: S1 Data — (ZIP) [file ppat.1013352.s011.zip › All_raw_data/Raw_data_Fig_9B_Anti-Flag_BtuB-TF-His+J-Flag.tif]

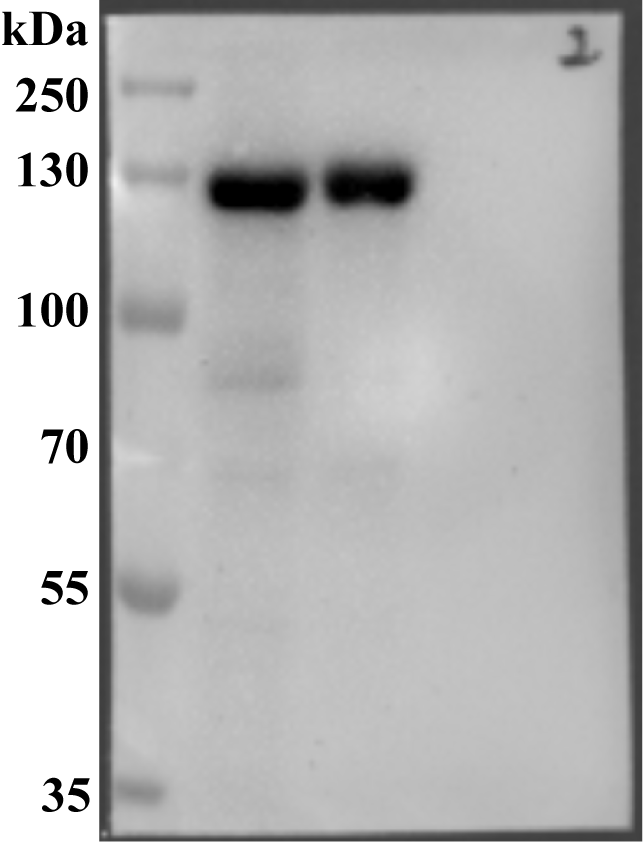

Supplement: S1 Data — (ZIP) [file ppat.1013352.s011.zip › All_raw_data/Raw_data_Fig_9B_Anti-His_BtuB-TF-His+J-Flag.tif]

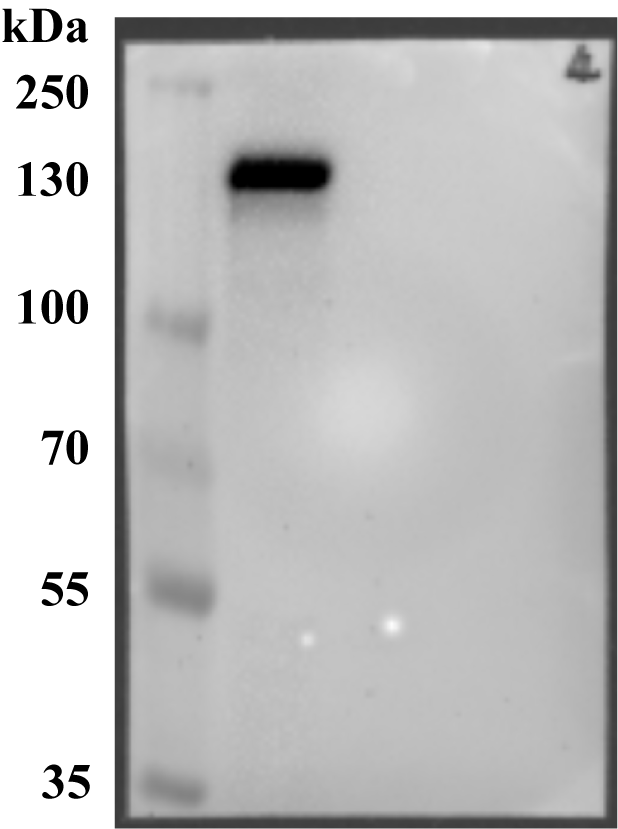

Supplement: S1 Data — (ZIP) [file ppat.1013352.s011.zip › All_raw_data/Raw_data_Fig_9C_Anti-Flag_OmpX-TF-His+J-Flag.tif]

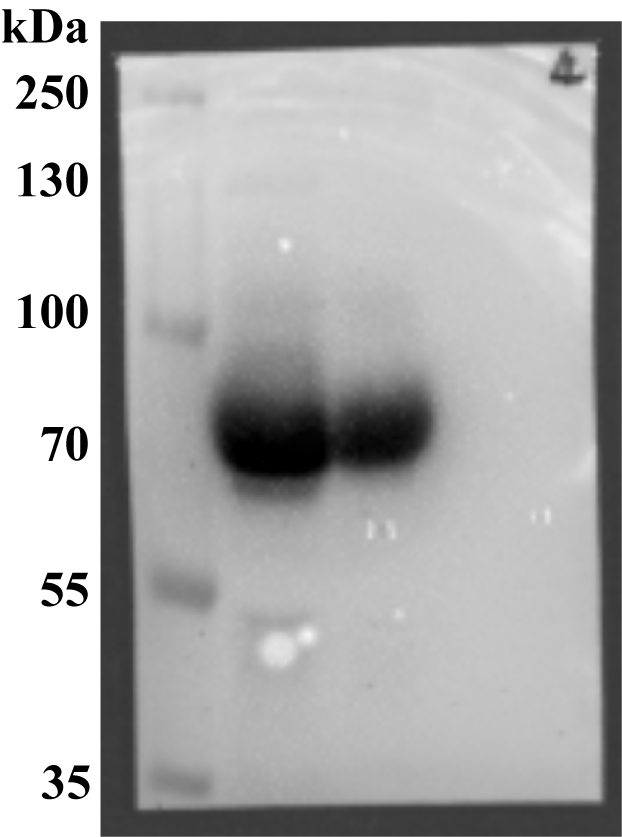

Supplement: S1 Data — (ZIP) [file ppat.1013352.s011.zip › All_raw_data/Raw_data_Fig_9C_Anti-His_OmpX-TF-His+J-Flag.tif]

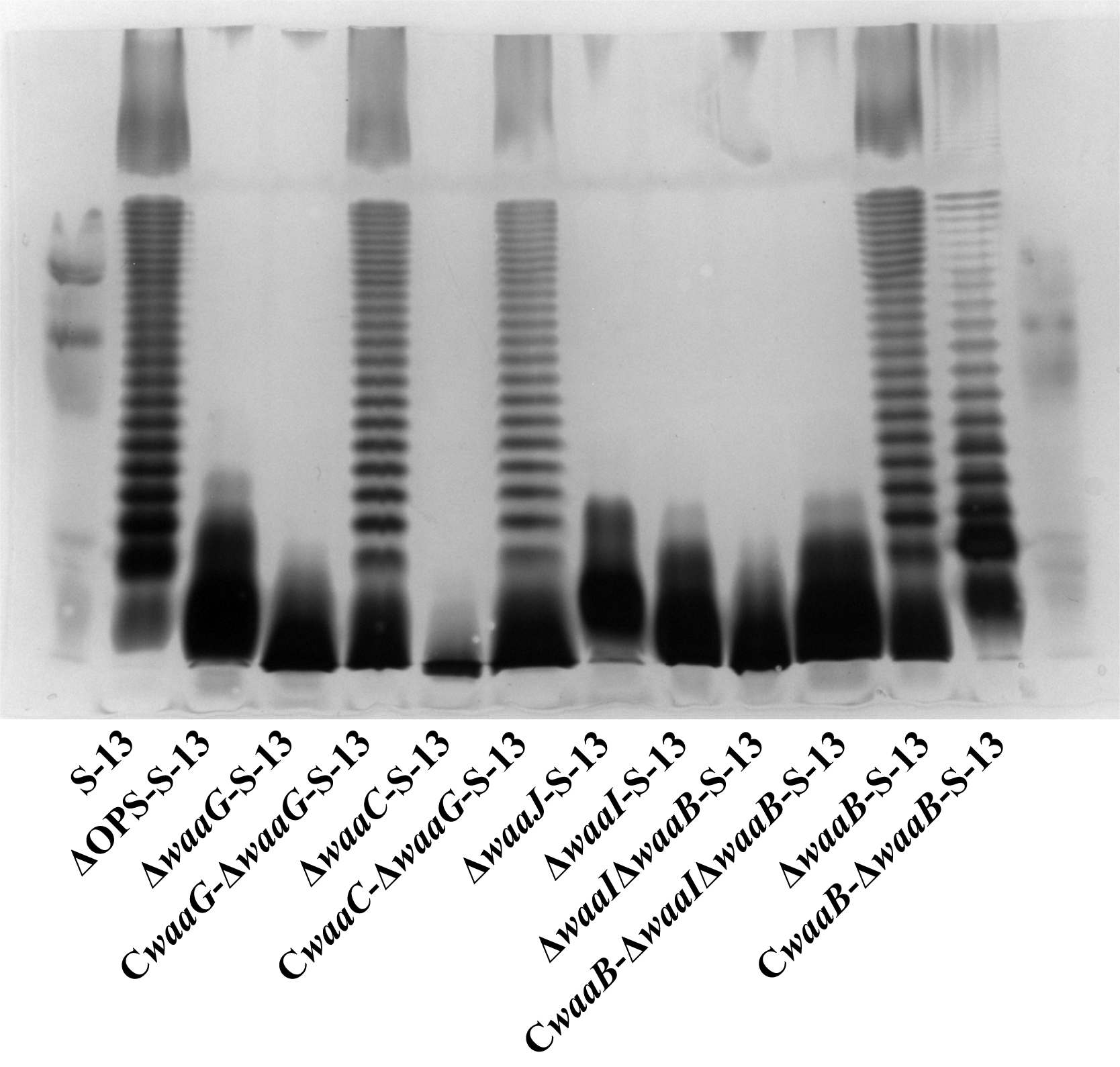

Supplement: S1 Data — (ZIP) [file ppat.1013352.s011.zip › All_raw_data/Raw_data_S1 Fig.tif]

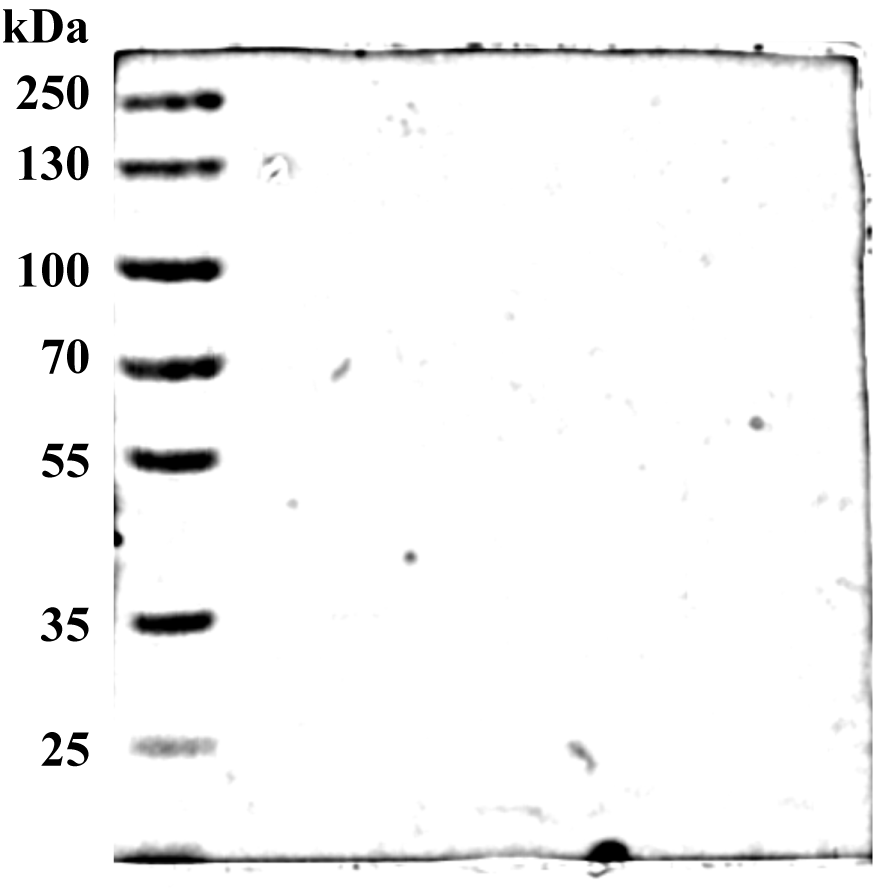

Supplement: S1 Data — (ZIP) [file ppat.1013352.s011.zip › All_raw_data/Raw_data_S6_Fig_Anti-Flag_TF-His+J-Flag.tif]

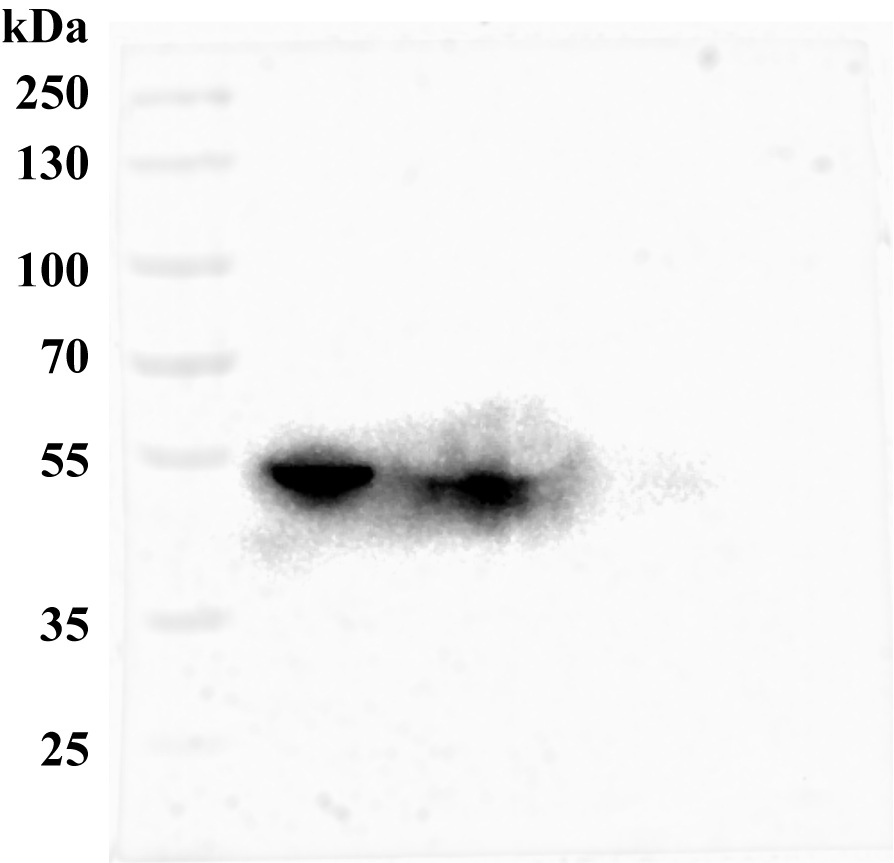

Supplement: S1 Data — (ZIP) [file ppat.1013352.s011.zip › All_raw_data/Raw_data_S6_Fig_Anti-His_TF-His+J-Flag.tif]
